# Regulation of distinct branches of the non-canonical Wnt-signaling network in *Xenopus* dorsal marginal zone explants

**DOI:** 10.1186/s12915-016-0278-x

**Published:** 2016-07-05

**Authors:** Veronika Wallkamm, Karolin Rahm, Jana Schmoll, Lilian T. Kaufmann, Eva Brinkmann, Jessica Schunk, Bianca Kraft, Doris Wedlich, Dietmar Gradl

**Affiliations:** Zoological Institute, Department of Cell and Developmental Biology, Karlsruhe Institute of Technology, 76131 Karlsruhe, Germany; Section Developmental Genetics, Institute for Human Genetics, University of Heidelberg, 69120 Heidelberg, Germany; Clinical Cooperation Unit Molecular Hematology/Oncology, German Cancer Research Center (DKFZ) and Department of Medicine V, University of Heidelberg, 69120 Heidelberg, Germany

**Keywords:** Wnt-signaling network, Convergent extension movements, Non-canonical Wnt-signaling

## Abstract

**Background:**

A tight regulation of the Wnt-signaling network, activated by 19 Wnt molecules and numerous receptors and co-receptors, is required for the establishment of a complex organism. Different branches of this Wnt-signaling network, including the canonical Wnt/β-catenin and the non-canonical Wnt/PCP, Wnt/Ror2 and Wnt/Ca^2+^ pathways, are assigned to distinct developmental processes and are triggered by certain ligand/receptor complexes. The Wnt-signaling molecules are closely related and it is still on debate whether the information for activating a specific branch is encoded by specific sequence motifs within a particular Wnt protein. The model organism *Xenopus* offers tools to distinguish between Wnt-signaling molecules activating distinct branches of the network.

**Results:**

We created chimeric Wnt8a/Wnt11 molecules and could demonstrate that the C-terminal part (containing the BS2) of Wnt8a is responsible for secondary axis formation. Chimeric Wnt11/Wnt5a molecules revealed that the N-terminus with the elements PS3-1 and PS3-2 defines Wnt11 specificity, while elements PS3-1, PS3-2 and PS3-3 are required for Wnt5a specificity. Furthermore, we used *Xenopus* dorsal marginal zone explants to identify non-canonical Wnt target genes regulated by the Wnt5a branch and the Wnt11 branch. We found that *pbk* was specifically regulated by Wnt5a and *rab11fip5* by Wnt11. Overexpression of these target genes phenocopied the overexpression of their regulators, confirming the distinct roles of Wnt11 and Wnt5a triggered signaling pathways. Furthermore, knock-down of *pbk* was able to restore convergent extension movements in Wnt5a morphants.

**Conclusions:**

The N-terminal part of non-canonical Wnt proteins decides whether the Wnt5a or the Wnt11 branch of the Wnt-signaling network gets activated. The different non-canonical Wnt branches not only regulate cellular behavior, but, surprisingly, also regulate the expression of different target genes. One of these target genes, *pbk*, seems to be the relevant target gene executing Wnt5a-mediated regulation of convergent extension movements.

**Electronic supplementary material:**

The online version of this article (doi:10.1186/s12915-016-0278-x) contains supplementary material, which is available to authorized users.

## Background

The Wnt-signaling network triggered by morphogens of the Wnt protein family is involved in numerous developmental processes. A recent milestone in the Wnt research field was the deciphering of the crystal structure of a Wnt/Fz complex [1]. Wnt molecules consist of 22 to 24 highly conserved cysteine residues important to establish the protein structure [2]. Figuratively, the Wnt ligand holds the Frizzled (Fz) receptor with its lipid modified thumb (binding site 1, BS1) and its index finger (binding site 2, BS2) in the pincer grip, with the thumb containing palmitoleic acid modification at the Ser187 and the index finger consisting of the cysteine-rich C-terminus [1]. Both binding sites are highly conserved. Additionally, Janda et al. [1] identified a third less conserved domain called pseudosite 3 (PS3). This PS3 is formed by three sequence motifs (PS3-1, PS3-2 and PS3-3) in the N-terminal region. The physiological relevance of this site is thus far unknown, but the authors speculate that it serves as a putative oligomerization motif [1].

Although the different Wnt proteins activate a complex signaling network, distinct branches of the network are assigned to specific functions [3]. The activation of the canonical Wnt/β-Catenin signaling pathway leads to the formation of the dorso-ventral axis [4]. Stabilized β-Catenin migrates into the nucleus, binds to the transcription factors TCF/LEF and regulates, as a transcriptional co-activator, the expression of numerous target genes. Wnt molecules that induce a secondary axis in *Xenopus* embryos [5] and transform C57MG cells [6] belong to the class of canonical Wnt ligands, whereas Wnt molecules that cannot induce a secondary axis and do not transform C57MG cells are so-called non-canonical Wnt ligands. Wnt1, Wnt3a and Wnt8 are representatives of canonical Wnt molecules, Wnt5a and Wnt11 are representatives of non-canonical Wnts. This separation into canonical and non-canonical Wnt proteins is challenged by the observation that, under certain circumstances, Wnt5a can also activate the Wnt/β-Catenin pathway and induce secondary axes in *Xenopus* [7]. However, for most cases, Wnt5a activates non-canonical Wnt pathways and activation of the Wnt/β-Catenin pathway by Wnt5a depends on the presence of Fz4 [8] or Fz5 [7].

Non-canonical, β-Catenin independent signaling pathways regulate stretching and narrowing of the dorso-ventral axis, a process termed convergent extension (CE) movements [9, 10]. These non-canonical pathways comprise the Wnt/Ca^2+^ [10], Wnt/PCP [11, 12], and Wnt/Ror2 [9] signaling pathways. Polarization and migration of mesodermal cells result in a medio-lateral narrowing and an anterior posterior elongation of the dorsal mesoderm [9, 13]. In *Xenopus*, the non-canonical Wnt proteins, xWnt5a and xWnt11, regulate different processes during CE movements in a non-redundant manner. At early gastrulation, xWnt11 triggers the polarization of the dorsal mesodermal cells [13]. Knock-down and overexpression of xWnt11 impairs cell polarization. As a consequence, explants of the dorsal marginal zone (DMZ) fail to elongate. In contrast, xWnt5a is responsible for cell migration of the polarized mesodermal cells towards the dorsal midline. Thus, DMZ explants of xWnt5a morphants and xWnt5a overexpressing embryos still elongate, but fail to constrict. Most of the cellular responses to a non-canonical Wnt signal are assigned to changes in the cytoskeleton and cell movements rather than to regulation of target gene expression. Indeed, in *Xenopus* paraxial protocadherin (PAPC) is the only xWnt5a target gene described so far [9].

In this study, we identified *pbk* as a novel xWnt5a target gene and *rab11fip5* as a novel xWnt11 target gene. Gain of function experiments revealed that pbk phenocopies xWnt5a overexpression and rab11fip5 phenocopies xWnt11 overexpression. Loss of function experiments demonstrate that both *rab11fip5* and *pbk* are relevant for proper CE movements. Epistasis experiments revealed that *pbk* is the xWnt5a target gene responsible for regulating CE movements. The analysis of non-canonical xWnt5a und xWnt11 chimeras demonstrates that the selective induction of the xWnt5a- and xWnt11-specific response relies on poorly conserved regions in the N-terminal domain. These regions do not overlap with the C-terminal region responsible for activating the canonical Wnt/β-Catenin signaling pathway.

## Results

One of the classical assays to distinguish canonical from non-canonical Wnt pathways is the axis duplication assay in *Xenopus*. To separate distinct non-canonical branches is more difficult, because the non-canonical Wnt ligands xWnt5a and xWnt11 both regulate CE movements during gastrulation of *Xenopus* [9]. These CE movements are the driving force for blastoporus closure and notochord extension [14]. Overexpression of both xWnt5a and xWnt11 results in misregulated blastoporus closure (data not shown). Accordingly, axial mesoderm is mislocated in a dose-dependent manner as shown by the localization of *chordin* expression (Additional file 1: Figure S1). Thus, neither the blastoporus closure nor the analysis of the axial mesodermal marker gene *chordin* provides a suitable read-out system to separate xWnt5a- and xWnt11-specific functions.

**Fig. 1:**
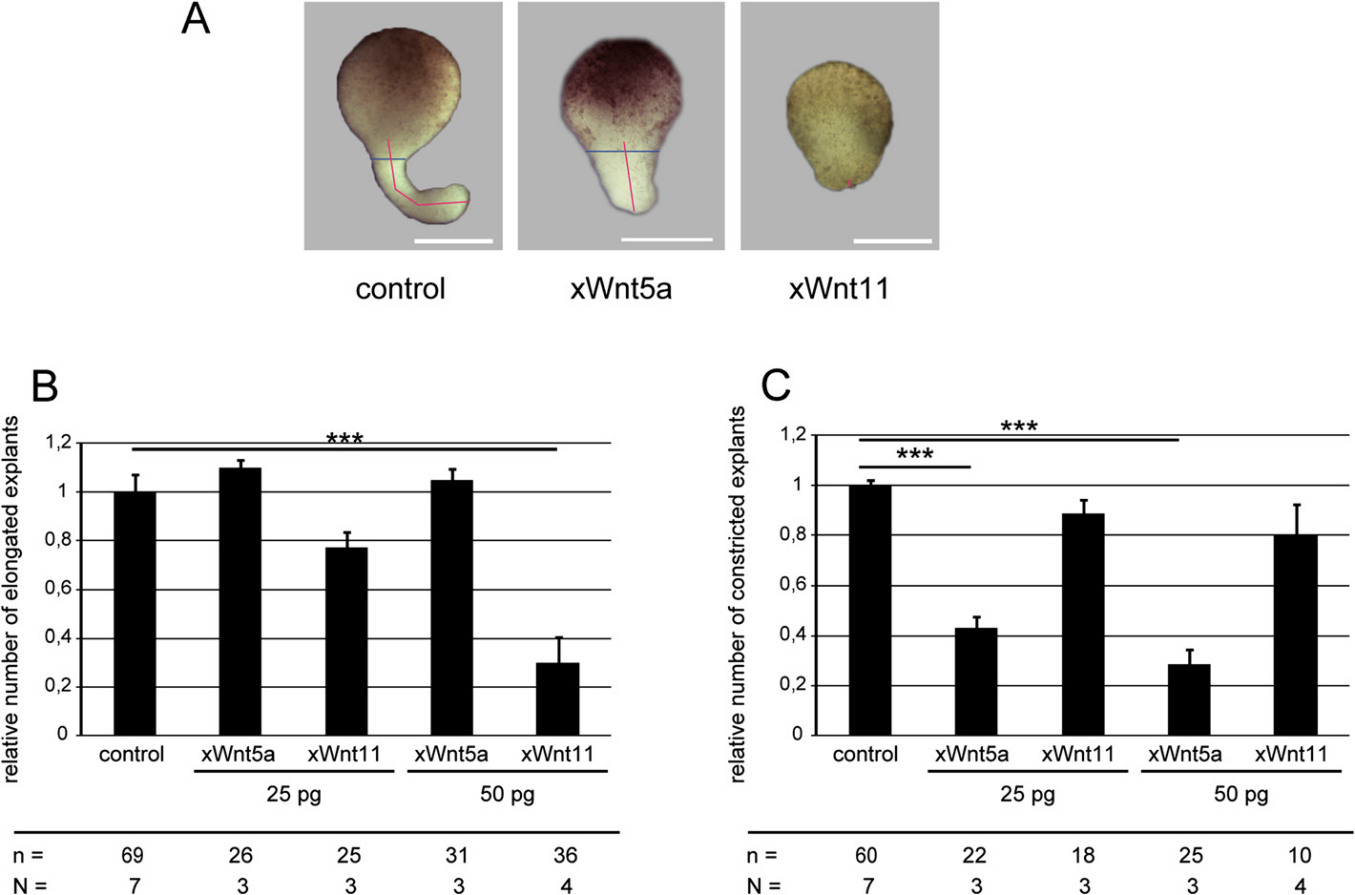
xWnt11 impairs elongation, xWnt5a impairs constriction. **a** Representative phenotypes of DMZ explants. The overexpression of xWnt5a results in broader, less elongated explants; the overexpression of xWnt11 inhibits elongation. Elongation and constriction were determined by phenotypically analyzing the length (red line) and width (blue line) of the outgrowth. Control explants show a protrusion with length > > width. For explants with inhibited elongation (xWnt11), hardly any protrusion can be detected. In explants showing failures in constriction, the protrusion is broader at the expense of length (xWnt5A). Constriction of explants that did not elongate could not be determined. **b** Quantification of elongation. xWnt5a has no influence on elongation, whereas xWnt11 inhibits elongation in a dose-dependent manner. **c** Quantification of constriction. xWnt5a inhibits constriction in a dose-dependent manner, xWnt11 has no significant influence on constriction of the explants. Shown is the frequency of the indicated phenotypes. The superimposed error bars illustrate the variation between N independent experiments (biological replicates). In each experiment, the absolute frequency of the indicated phenotypes was normalized to the control siblings. In total, 69 explants of the uninjected controls could be analyzed, 60 of which elongated and could be evaluated for constriction. For 50 pg xWnt11 this means that n = 36 explants were analyzed for “relative elongation”, but only n = 10 explants could be considered to analyze relative constriction. N: number of biological replicates; n: number of analyzed explants. *** *P* < 0.001 according to Fisher’s exact test; Bars: 200 μm

DMZ explants, which autonomously undergo CE movements, provide a more suitable analysis system [15]. The morphology of these explants allows analysis of elongation and constriction separately. While xWnt11 is required early in CE for the polarization of the dorsal mesodermal cells [13], xWnt5a is later responsible for the cell migration of the polarized mesodermal cells towards the dorsal midline. Consistently, functional differences between xWnt5a and xWnt11 also became phenotypically obvious in our study: xWnt5a mainly blocks constriction, xWnt11 blocks elongation (Fig. 1a). Wild-type explants form a long slim protrusion (red line in Fig. 1a), with a tissue constriction at the level of the former upper blastopore lip (blue line in Fig. 1a). Misregulation of the constriction process results in a different shape of the explants. The tissue protrusion is broader at the expense of length and tissue constriction is less prominent (examples: xWnt5A, Fig. 1a, 200 pg Chimera 3.2 Fig. 3, 250 pg pbk in Fig. 7c). When the elongation process is blocked, no tissue protrusion is formed, and a constriction cannot be observed (examples: xWnt11, Fig. 1a 50 and 200 pg Chimera 1.2 in Additional file 2: Figure S5). For evaluation, DMZ explants were assigned to these three categories. We determined the frequency for elongation (all explants) and constriction (only elongated explants) normalized to DMZ explants of control siblings, thereby taking into consideration that non-elongated explants fail in evaluating constriction. Almost all xWnt5a overexpressing DMZ explants elongated, whereas their constriction was significantly inhibited in a dose-dependent manner (Fig. 1b,c). Overexpression of xWnt11, instead, had a significant and dose-dependent influence on the elongation of the explants. Constriction of the elongated explants remained unaffected (Fig. 1b,c). Thus, in DMZ explants, the effects mediated by xWnt5a and xWnt11 are clearly distinguishable. Therefore, DMZ explants provide a powerful tool to analyze the mechanisms and consequences of specifically activating the xWnt5a and xWnt11 branches of the non-canonical Wnt-signaling network.

To identify regions in the Wnt proteins responsible for the selective activation of distinct branches we designed a set of chimeras consisting of parts of the xWnt11 and xWnt8a sequences and parts of the xWnt5a and xWnt11 sequences, respectively. To fuse the sequences of the different Wnt molecules we accorded to the crystal structure of Wnt8/FzCRD [1] and retained Janda’s nomenclature for the different domains.

First, we investigated which part of the Wnt ligands is responsible for the activation of the canonical Wnt branch. As a read-out system we chose the *Xenopus* secondary axis assay. The chimera between canonical xWnt8a and non-canonical xWnt11 was fused between the BS1 and BS2 (Additional file 3: Figure S2A). The translation of the fusion constructs was verified with an in vitro combined transcription and translation assay before performing the secondary axis assays (Additional file 4: Figure S3A). As expected, ventral injection of xWnt8a led to a robust induction of secondary axes, whereas the injection of xWnt11 had no effect. Only the chimera with the C-terminal part of xWnt8a was able to induce a secondary axis. However, compared to wild-type xWnt8a, the chimera was less efficient because, for secondary axis induction, the mRNA dose had to be highly increased. The chimera containing the C-terminal part of xWnt11 was not able to induce a secondary axis. Thus, consistent with earlier data showing axis induction by an xWnt5a/8a chimera [16], we identified the C-terminus including BS2 as the region responsible for activating the canonical Wnt pathway.

To decipher whether the same region is also responsible for the activation of non-canonical Wnt pathways we tested these chimeras in the elongation assay (Additional file 5: Figure S4). Interestingly, the same construct that induced a secondary axis also inhibited the elongation of DMZ explants. Again, the effect was mild compared to the wild-type Wnt, in this case xWnt11. However, our results indicate that canonical Wnt signaling is mediated mainly by the C-terminal BS2, whereas activation of non-canonical Wnt signaling tends to be mediated by the N-terminal structures of the ligand. It is worth noticing that none of the explants showed a short and broad protrusion. Thus, constriction remained unaffected.

**Fig. 2:**
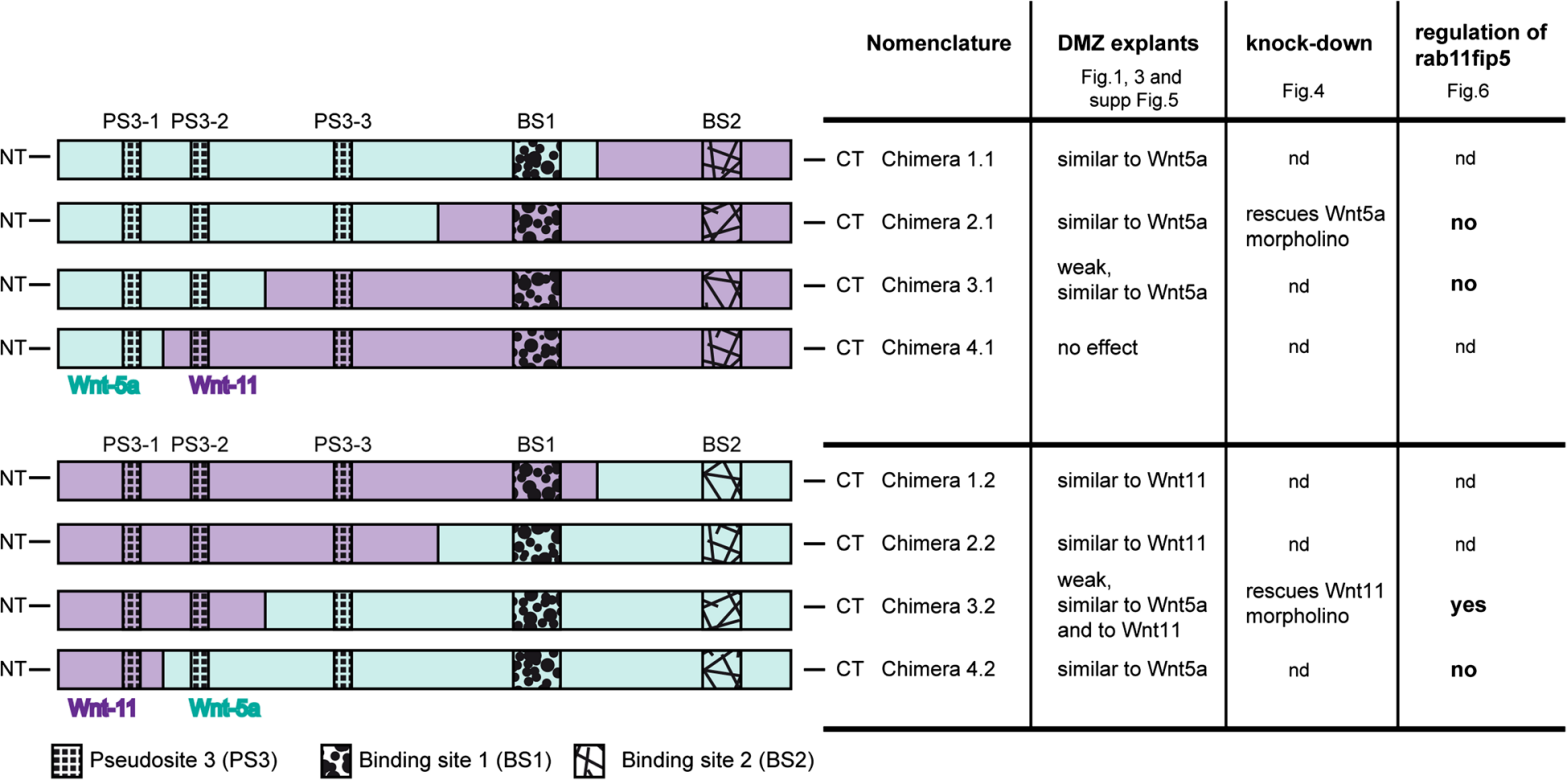
Summarizing scheme of Wnt5a/Wnt11 chimeras and their effects

In order to identify the regions responsible for xWnt5a and xWnt11 signaling we created a set of xWnt5a/11 chimeras and used DMZ explants as a read-out system (Fig. 2). As for the xWnt8a/11 chimeras, we generated constructs exchanging the C-terminal domain containing the BS2 of xWnt5a and xWnt11, respectively. Furthermore, we successively exchanged a larger portion of the C-terminus in more fusion constructs. To simplify the nomenclature, the fusion sites were numbered consecutively (1–4), whereby N-terminal xWnt5a fusions are termed X.1 and N-terminal xWnt11 fusions are termed X.2 (Fig. 2). All chimeras were transcribed in vitro (Additional file 4: Figure S3B) and activated the non-canonical ATF2-luciferase reporter, which monitors non-canonical Wnt pathway activation (Additional file 4: Figure S3C). Furthermore, all chimeras interfered with blastopore closure and misplaced the expression of the mesodermal marker gene *chordin* (data not shown). Thus, all chimeras fulfill the criteria for functional non-canonical Wnt molecules and were used for further investigation in DMZ explants. We injected 50 pg and 200 pg mRNA of each chimera; 50 pg was found to be the optimal dose to distinguish the effects of wild-type xWnt5a and xWnt11 in the elongation assay (Fig. 1).

To establish chimera pair 1, the C-terminus including BS2 was exchanged. The Wnt5a/Wnt11 chimera 1.1 turned out to induce the same phenotype as xWnt5a – it did not affect elongation but significantly impaired constriction in a dose-dependent manner. The Wnt11/Wnt5a chimera 1.2, instead, phenocopied xWnt11 and inhibited elongation (Additional file 2: Figure S5A–C). Therefore, both chimeras retained the properties of their N-terminal part and the region providing the individual non-canonical Wnt with its subtype-specific properties is located in the BS1 and/or PS3.

To create chimera pair 2, the Fz binding domains (BS1 and BS2) of xWnt5a and xWnt11 were separated from the regions contributing to PS3 (Fig. 2). Both of these chimera pairs induced a similar phenotype – they significantly inhibited elongation and constriction. Thus, parts of the specificity towards activation of a distinct non-canonical branch got lost. However, the chimera with PS3 of xWnt5a (chimera 2.1) still had a stronger effect on constriction, and thus phenocopied xWnt5a, and the chimera with PS3 of xWnt11 (chimera 2.2) still had a stronger effect on elongation, and thus phenocopied xWnt11 (Additional file 2: Figure S5D-E). This leads to the assumption that the domain around the BS1 and the domains constituting the PS3 are involved in the specification of the Wnt molecules. Since BS1 consists of the highly conserved fatty acid modified region important for Fz binding we focused our analyses on the influence of the poorly characterized PS3.

**Fig. 3:**
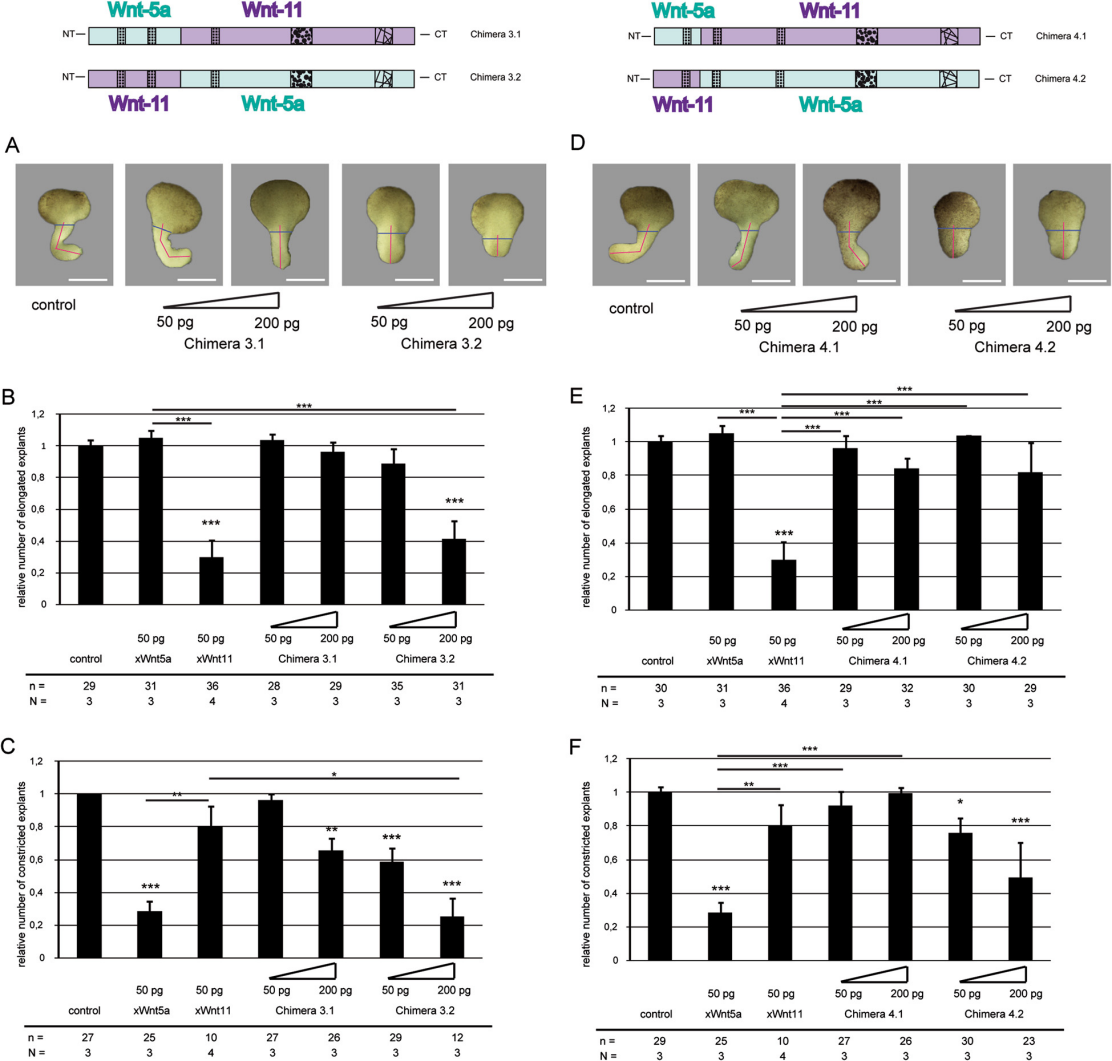
Analysis of chimera pair 3 and 4 in dorsal marginal zone (DMZ) explants. **a** Representative phenotypes of DMZ explants of embryos injected with the indicated mRNAs of chimera pair 3. Wnt5a/Wnt11 Chimera 3.1 inhibits constriction, whereas Wnt11/Wnt5a chimera 3.2 influences constriction and elongation. **b** Quantification of elongation. **c** Quantification of constriction. **d** Representative phenotypes of DMZ explants of embryos injected with the indicated mRNAs of chimera pair 4. Wnt5a/Wnt11 chimera 4.1 does not disturb convergent extension movements, whereas Wnt11/Wnt5a chimera 4.2 inhibits constriction. **e** Quantification of elongation. **f** Quantification of constriction. Shown is the frequency of the indicated phenotypes. The superimposed error bars illustrate the variation between N independent experiments. In each experiment, the absolute frequency of the indicated phenotypes was normalized to the control siblings. N: number of biological replicates, n: number of analyzed explants, *** *P* < 0.001, ** *P* < 0.01, * *P* < 0.05 according to Fisher’s exact test, Bars: 200 μm

To create chimera pair 3, xWnt5a and xWnt11 were fused in a conserved domain between PS3-2 and PS3-3 (Fig. 2). The Wnt5a/Wnt11 chimera 3.1 had no influence on elongation. Instead, constriction of DMZ explants was significantly inhibited compared to the control. However, compared to Wnt11, we found no significant difference. Our data indicate that overexpression of this chimera induced a weak xWnt5a-like phenotype. The Wnt11/Wnt5a chimera 3.2, instead, displayed both phenotypes, elongation was inhibited and the elongated explants appeared less constricted (Fig. 3a–c). This leads to the assumption that, for activation of the xWnt5a branch the PS3-3 is necessary, but for activation of the xWnt11 branch the domains around PS3-2 and PS3-1 are necessary.

In chimera pair 4, the N-terminal PS3-1 was exchanged. The Wnt5a/Wnt11 chimera 4.1 had no impact on CE movements. The explants displayed wild-type morphology. The Wnt11/Wnt5a chimera 4.2 significantly disturbed constriction (Fig. 3d,f). Thus, this construct phenocopied xWnt5a.

**Fig. 4:**
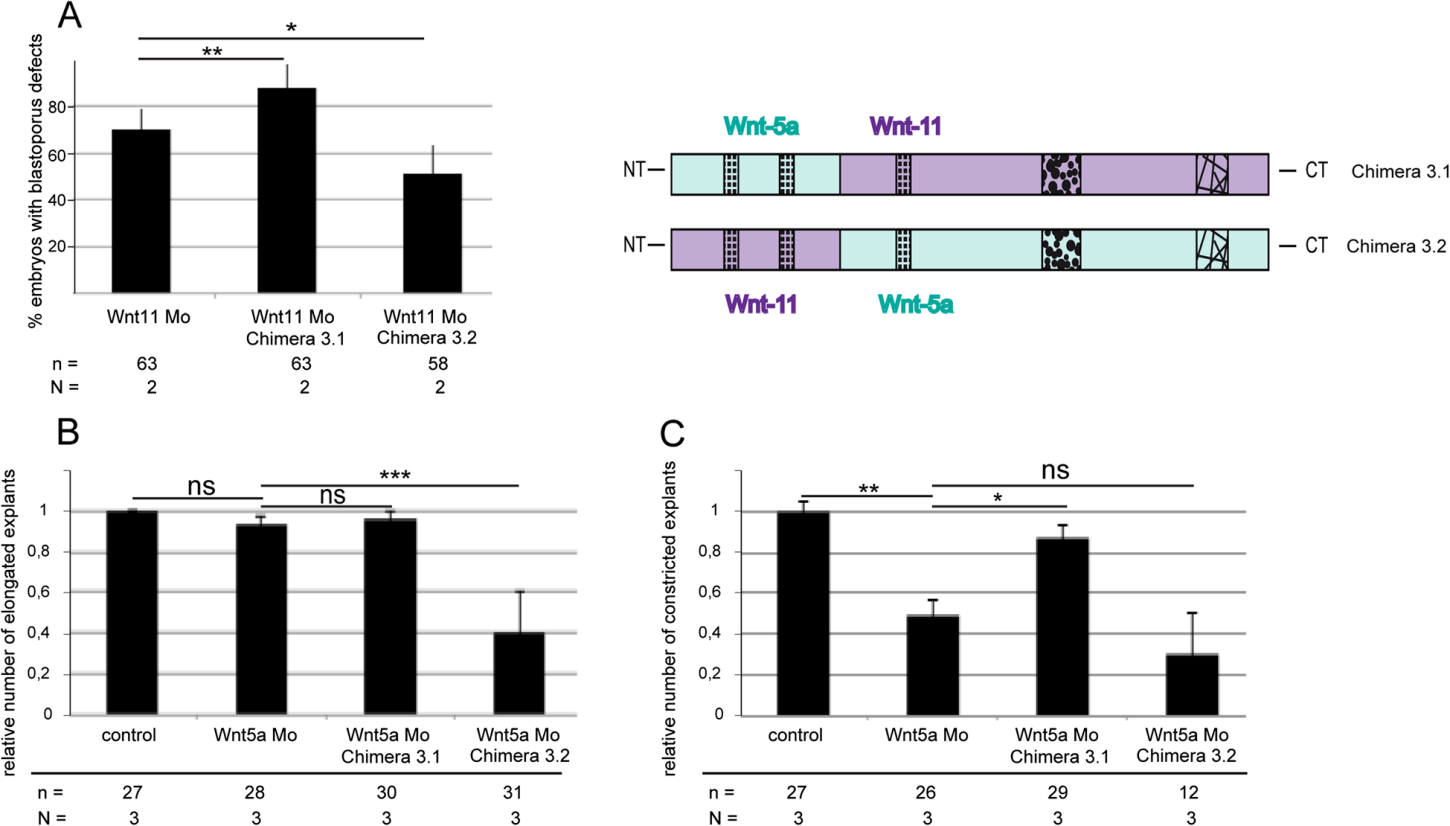
Rescue experiments chimera. To test whether the chimeric constructs can compensate for the loss of xWnt11 and xWnt5a, 200 pg of Wnt5a/Wnt11 chimera 3.1 and Wnt11/Wnt5a 3.2 were co-injected with 2.5 pmol of an xWnt11 (Wnt11 Mo) and xWnt5a (Wnt5a Mo) morpholino (Mo) antisense oligonucleotide. **a** To determine the effect of the Wnt11 Mo, we counted the fraction of stage 12 embryos with open blastopore (blastopore defects). To test whether the chimeric constructs can compensate for the loss of xWnt5a we calculated (**b**) relative elongation and (**c**) relative constriction of dorsal marginal zone explants. Shown is the frequency of the indicated phenotypes. The superimposed error bars illustrate the variation between N independent experiments. N: number of biological replicates, n: number of analyzed explants, *** *P* < 0.001, ** *P* < 0.01, * *P* < 0.05 according to the χ^2^ test (**a**) and Fisher’s exact test (**b**, **c**)

The overexpression of the chimera revealed that, for activating the Wnt11 branch, the very N-terminus including PS3-2 and PS3-1 is necessary, whereas the specificity for the Wnt5a branch additionally includes PS3-3. To prove this finding we tested, in reconstitution experiments, whether a chimera can compensate for the loss of endogenous xWnt5a and xWnt11. We expected that the chimera with the N-terminal part of xWnt11, including PS3-1 and PS3-2 (chimera 3.2), can compensate for the loss of xWnt11. Indeed, this chimera restored blastopore closure in xWnt11 morphants (Fig. 4a). Interestingly, this chimera could not replace xWnt5a. Most of the explants expressing chimera 3.2 in a Wnt5a morphant background failed to elongate (Fig. 4b) and most of the elongated explants failed to constrict (Fig. 4c). Since chimera 3.2 contains PS3-3 of xWnt5a, this means that PS3-3 is not sufficient for Wnt5a signaling. The Wnt5a/Wnt11 chimera 3.1, instead, could compensate for the loss of xWnt5a (Fig. 4c), but not for the loss of xWnt11 (Fig. 4a).

Taken together, the analyses of chimeras revealed that, for activation of the distinct branches of the Wnt-signaling network, different regions in the proteins are responsible. To activate non-canonical Wnt branches, the N-terminal part is essential. Herein, a region ranging from PS3-1 to PS3-3 preferentially activates the xWnt5a branch, the regions referred to as PS3-1 and PS3-2 preferentially activate the xWnt11 branch. The decision between canonical and non-canonical Wnt-signaling relies on the C-terminal part. A C-terminus of a “canonical” Wnt is necessary and sufficient to convert a non-canonical Wnt into a canonical one and to activate the canonical Wnt branch.

The highly specific response of the involuting mesoderm to xWnt5a (no constriction) and xWnt11 (no elongation) (Fig. 1) prompted us to ask whether these non-canonical Wnt branches regulate specific sets of target genes. Therefore, we performed a comparative transcriptome analysis of DMZ explants derived from xWnt5a and xWnt11 morphants and control morpholino-injected siblings. The explants were grown until sibling embryos reached stage 12, a stage when the cells of the dorsal mesoderm are bipolar and start to migrate towards the dorsal midline.

**Fig. 5:**
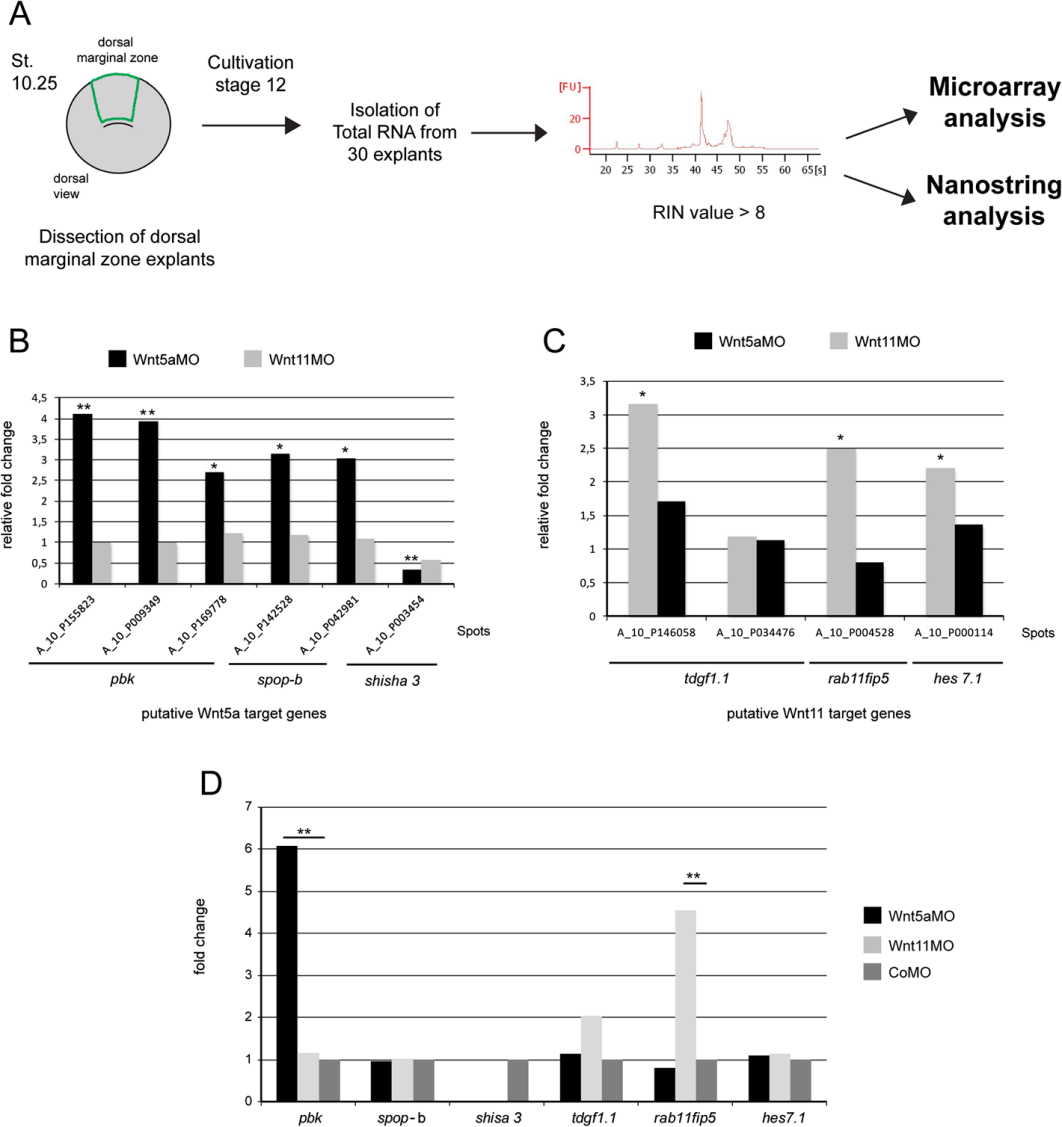
*pbk* and *rab11fip5* are specific non-canonical Wnt target genes. **a** Dorsal marginal zone (DMZ) explants were dissected at stage 10.25 and cultivated until siblings reached stage 12. Total RNA was isolated from 30 DMZ explants. Samples with an RNA integrity number value > 8 were analyzed in a microarray or nanostring analysis. **b** Differential regulation of six spots on the microarray representing three putative xWnt5a target genes. Shown is the average fold change of biological triplicates, the indicated *P* value is relative to the control morphant-injected explants, ** *P* < 0.01, * *P* < 0.05. **c** Differential regulation of four spots on the microarray representing three putative xWnt11 target genes. Shown is the average fold change of biological triplicates (**d**). Reevaluation of the six putative non-canonical Wnt target genes by nanostring analysis. Only *pbk* and *rab11fip5* were found to be differentially regulated. Shown is the average fold change of biological triplicates. ** *P* < 0.01 according to one-sample *t* test

Total RNA was extracted from 30 stage 12 DMZ explants (Fig. 5a). The comparative transcriptome analysis was performed in three independent biological replicates on a *Xenopus* 4 × 44 K gene expression microarray chip from Agilent (Atlas Biolabs, Germany). Candidates with more than 2-fold difference compared to the control (*P* < 0.05) were considered as putative target genes. For xWnt5a, only 67 spots on the array fulfilled these criteria (Additional file 6: Table S1), for xWnt11 we identified 148 spots (Additional file 7: Table S2), among which 15 were regulated by both ligands. The overall low number of genes regulated by the non-canonical Wnts xWnt5a and xWnt11 might be explained by the fact that, in contrast to canonical Wnt signaling, non-canonical Wnt pathways have only mild impact on transcriptional regulation [17]. Indeed, a parallel screen with DMZ explants derived from xLef-1 morphants identified almost 700 differentially regulated spots (data not shown).

From the putative non-canonical Wnt target genes we selected PDZ binding kinase/T-cell originated protein kinase (PBK/TOPK, short *pbk*), speckle type POZ protein (*spop-b*), and *shisa 3* as the most interesting candidates specifically regulated by xWnt5a (Fig. 5b), and RAB family interacting protein 5 (*rab11fip5*), hairy and enhancer of split 7 gene 1 (*hes7.1*), and teratocarcinoma-derived growth factor 1 (*tdgf1.1*) specifically regulated by xWnt11 (Fig. 5c) for further analyses. In a nanostring analysis the transcript number of the putative target genes was counted in control morphant (CoMO), xWnt5a morphant (Wnt5aMO), and xWnt11 morphant (Wnt11MO) stage 12 DMZ explants. Among the six candidates, only two were indeed regulated in a Wnt dependent manner (Fig. 5d). For *pbk* we found six times more transcripts in the xWnt5a morphant background compared to the CoMO-injected DMZ explants (Fig. 5d). In the xWnt11 morphants, the expression of *pbk* remained unchanged. *rab11fip5* transcripts, instead, were four-fold enriched in the xWnt11 morphants but remained unchanged in the xWnt5a morphants. Thus, with *rab11fip5* and *pbk* we provide here the first evidence that different branches of the non-canonical Wnt-signaling network specifically regulate the expression of different target genes – xWnt5a regulates the expression of *pbk* and xWnt11 regulates the expression of *rab11fip5*.

**Fig. 6:**
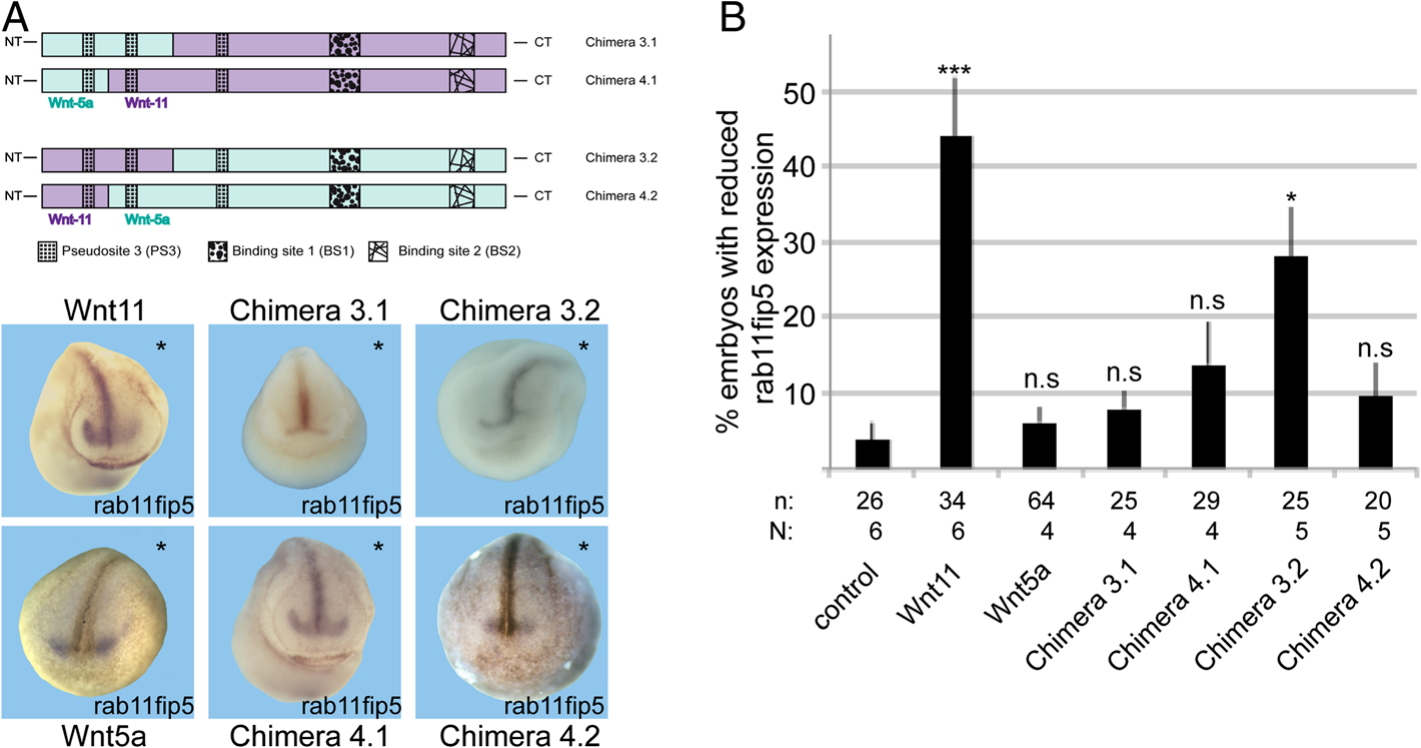
The N-terminal part of Wnt11 is required to suppress *rab11fip5* expression. 200 pg of the indicated Wnt mRNA were co-injected with the lineage tracer Dextran-FITC into one blastomere of two-cell stage embryos. **a** At the neurula stage, *rab11fip5* expression was determined by RNA in situ hybridization. Asterisks mark the injected site. **b** The quantification of the phenotype. Shown is the absolute frequency of the indicated phenotypes. The superimposed error bars illustrate the variation between N biological replicates, n: number of embryos. * *P* < 0.05, *** *P* < 0.001 according to the χ^2^ test

We amplified the open reading frame of *pbk* and *rab11fip5* from gastrula stage embryos and from DMZ explant cDNA. Expression analyses (Additional file 8: Figure S6) revealed that both non-canonical Wnt targets are maternally enriched in the animal hemisphere. During the gastrula stage, both genes are uniformly expressed in the whole embryo but still appear enriched in the animal hemisphere. From the neurula stage onwards, *pbk* is expressed in the dorsal part of the developing CNS, whereas *rab11fip5* is expressed in the very anterior CNS and in a ring surrounding the cement gland (Additional file 8: Figure S6). We used these novel target genes to confirm that the N-terminal part of non-canonical Wnts is relevant for selective pathway regulation. Therefore, we injected mRNA encoding for xWnt11, xWnt5a and a subset of our chimera into one blastomere of two-cell stage embryos in order to see the effect in direct comparison with the uninjected control side. While we did not observe effects of overexpressed xWnt5a on *pbk* expression (not shown), overexpression of xWnt11 led to a drastic decrease of *rab11fip5* at the injected side in 40 % of the injected embryos (Fig. 6). Thus, xWnt11 is necessary and sufficient to down regulate *rab11fip5* expression, whereas xWnt5a is necessary but not sufficient to down regulate *pbk* expression. As expected, the chimera 3.2 with the N-terminal part of xWnt11, including PS3-1 and PS3-2, also significantly reduced *rab11fip5* expression, whereas the chimeras with the N-terminal part of xWnt5a did not (Fig. 6).

**Fig. 7:**
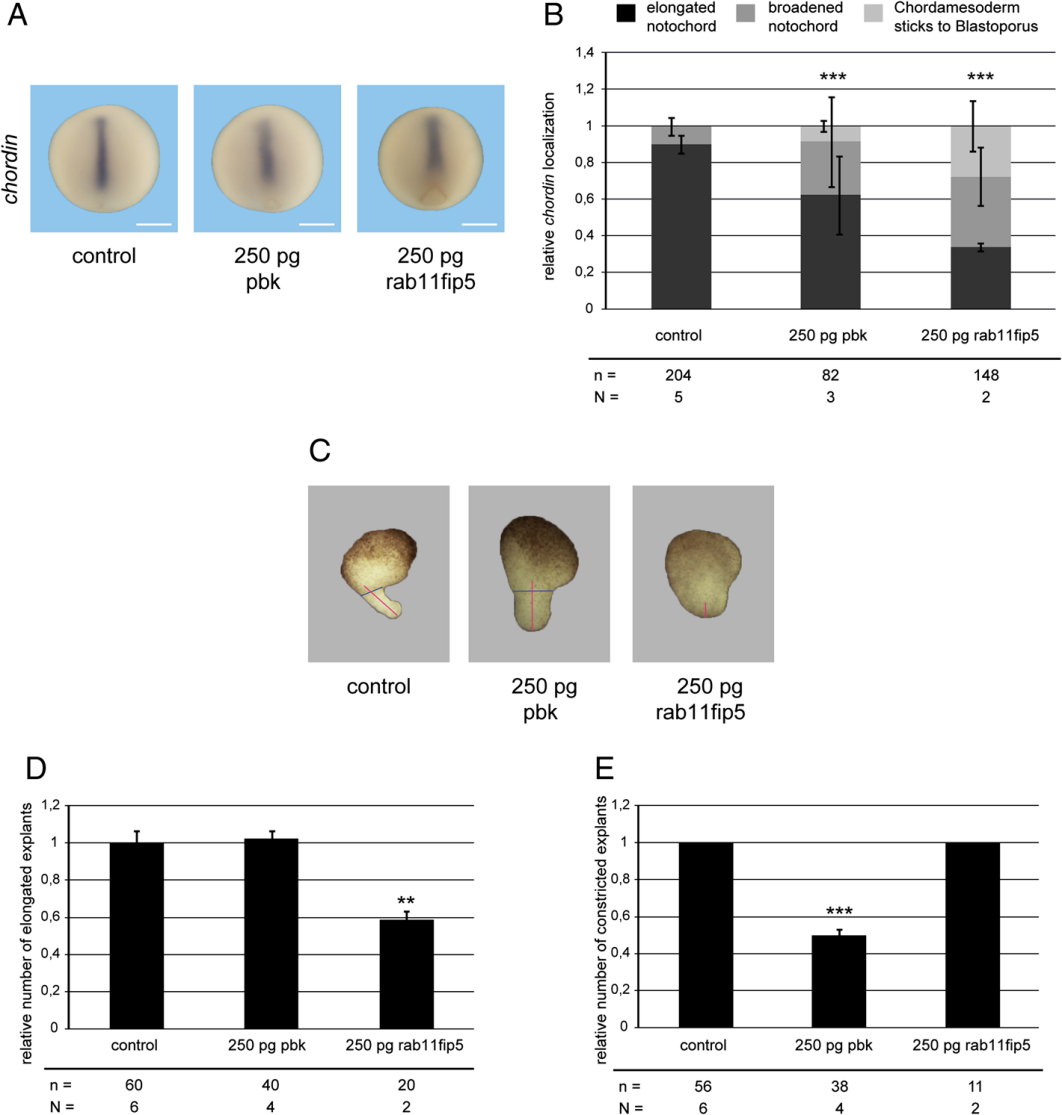
Pbk and rab11fip5 interfere with convergent extension movements. **a** Representative whole mount in situ hybridization for *chordin* of stage 12 embryos injected dorsal equatorially at the 4-cell stage with the indicated mRNAs. The dorsal overexpression of pbk and rab11fip5 results in a shorter and broader *chordin* expression. Bars: 500 μm. **b** Quantification of *chordin* phenotypes. *** *P* < 0.001 according to χ^2^ significance test (**c**). Phenotypes of dorsal marginal zone (DMZ) explants derived from pbk and rab11fip5 overexpressing embryos. Overexpression of pbk leads to broader elongated explants and overexpression of rab11fip5 results in an inhibition of elongation. Bars: 200 μm. **d** Quantification of elongation. Pbk does not interfere with elongation but rab11fip5 significantly inhibits elongation. **e** Quantification of constriction. Pbk overexpressing DMZ explants fail to constrict whereas rab11fip5 does not affect constriction. Shown is the frequency of the indicated phenotypes. In each experiment, the absolute frequency of the indicated phenotypes was normalized to the control siblings. The superimposed error bars illustrate the variation between N independent experiments. ** *P* < 0.01, *** *P* < 0.001 according to Fisher’s exact test. N: number biological replicates, n: number of analyzed embryos/explants

To decipher whether the novel xWnt5a- and xWnt11-specific target genes indeed regulate CE movements we overexpressed them in the DMZ. The overexpression of both target genes resulted in a delay in blastopore closure (data not shown) and an altered *chordin* expression pattern (Fig. 7a, b) reminiscent to the overexpression of xWnt5a and xWnt11. This indicates that the non-canonical Wnt target genes are indeed involved in the migration of the dorsal mesoderm during CE movements and not in mesoderm induction. For a more specific characterization, we again analyzed the elongation and constriction of DMZ explants. Overexpression of 250 pg rab11fip5 inhibited elongation and overexpression of 250 pg pbk inhibited constriction (Fig. 7c,e). Thus, the xWnt5a-specific target gene *pbk* phenocopies xWnt5a and the xWnt11-specific target gene *rab11fip5* phenocopies xWnt11.

To test whether the target genes *pbk* and *rab11fip5* join the long list of feedback target genes (http://web.stanford.edu/group/nusselab/cgi-bin/wnt/) we analyzed the activation of the non-canonical Wnt target promoter ATF2-luciferase [18]. xWnt5a activated the ATF2 reporter 7-fold and xWnt11 6-fold. Neither *pbk* nor *rab11fip5* regulated the ATF2 reporter or influenced the xWnt5a- and xWnt11-mediated ATF2-luciferase activation (Additional file 9: Figure S7A,B). Therefore, our novel non-canonical Wnt target genes are not feedback regulators.

**Fig. 8:**
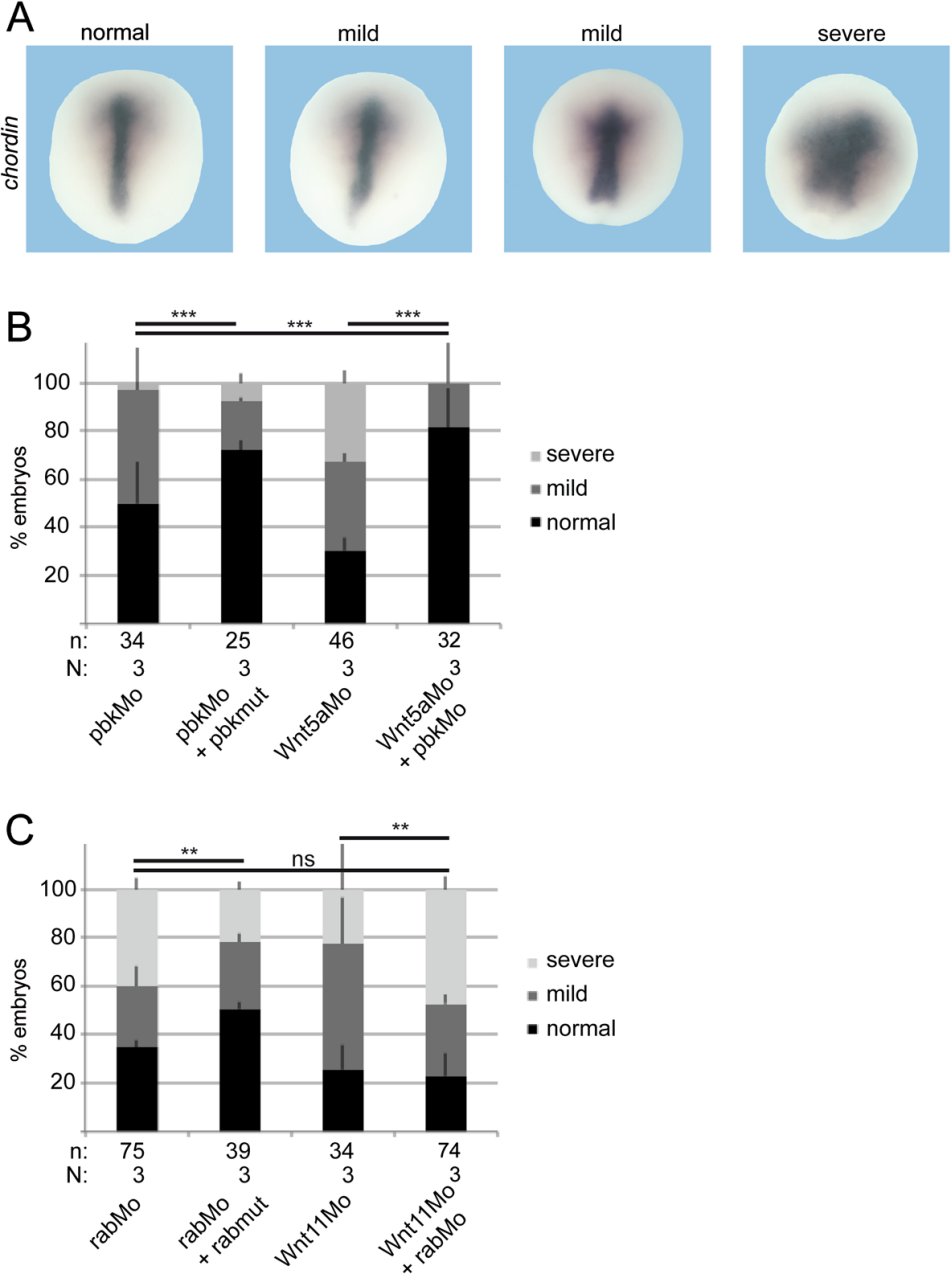
Epistasis experiments. Injection of antisense morpholinos specific for xWnt5a (Wnt5aMo), xWnt11 (Wnt11Mo), pbk (pbkMo), and rab11fip5 (rabMo) impaired convergent extension movements as seen as mild phenotype by a shortened and broad *chordin* expression or a strong phenotype by a blurred chordin expression or a staining at the borders of the non-closing blastoporus. **a** Shows representative dorsal marginal zone explants. **b** Quantification of the epistasis experiments revealed that knock-down of pbk can compensate for the loss of xWnt5a, but (**c**) knock-down of rab11fip5 cannot compensate for the loss of xWnt11. Shown is the absolute frequency of the indicated phenotypes. The superimposed error bars illustrate the variation between N biological replicates. ** *P* < 0.01, *** *P* < 0.001 according to the χ^2^ test, n: number of analyzed embryos

To test whether endogenous *pbk* and *rab11fip5* are necessary for CE movements we knocked down their expression through the morpholino approach (Additional file 10: Figure S8A). Indeed, the *pbk* morpholino induced mislocalization of *chordin* in a dose-dependent manner. This mislocalization ranged from a mild phenotype with *chordin* localized in a short and broad stripe (Fig. 8a), to a severe phenotype with blurred *chordin* expression (Fig. 8a) or even a ring of *chordin* around the open blastopore (Additional file 10: Figure S8C). Normal *chordin* expression in the *pbk* morphants was partially restored by co-injection of a *pbk* rescue construct (Fig. 8b), which is not targeted by the morpholino (Additional file 10: Figure S8A). Moreover, knock-down of *pbk* partially restored normal *chordin* expression in xWnt5a morphants (Fig. 8b). This means that *pbk* is not only a xWnt5a-specific non-canonical target, but also acts as main effector of xWnt5a regulated CE movements.

Similar to *pbk*, the effects of the *rab11fip5* morpholino could also be compensated by co-injection of a rescue construct (Fig. 8c), which is not targeted by the morpholino (Additional file 10: Figure S8A). However, in contrast to the Wnt5a/pbk pair, no recue was observed in the Wnt11/rab11fip5 double morphants. Instead, both morpholinos seem to induce mislocalization of *chordin* in an additive manner (Fig. 8c and Additional file 10: Figure S8C). Thus, the Wnt11 target gene *rab11fip5* is not the main executer of Wnt11 and also displays Wnt11 independent functions during CE movements.

## Discussion

With the detailed analysis of chimeric Wnt molecules in the model organism *Xenopus* we show, for the first time, that specific domains within Wnt molecules determine the activation of distinct branches of the Wnt-signaling network. We chose chimeras instead of deletion constructs since the cysteines, which are responsible for the ternary structure, are distributed over the entire Wnt molecule. As soon as only a few cysteines are missing or misplaced the folding is disturbed and the resulting Wnt proteins are biologically inactive [2].

The canonical Wnt branch only gets activated if the chimera contains the C-terminus of a canonical Wnt, which is in line with earlier reports [16]. The C-terminus of xWnt8a comprises the Fz BS2 and the less conserved linker region. The BS2 is the highly conserved interaction domain for the Fz CRD [1]. Recruitment of different Fz subtypes might be responsible for the decision of whether canonical or non-canonical Wnt-signaling pathways get activated. For mini Wnt8a (90 C-terminal amino acids) a higher affinity to Fz8 than to Fz5 has been described [1, 19]. The linker region adjacent to the BS2 mediates Lrp binding and thus recruitment of the co-receptor necessary for full activation of the canonical Wnt branch [20, 21]. Possibly, the decision to activate the canonical Wnt pathway relies on both recruitment of a specific (subset of) Fz receptor(s) via BS2 and recruitment of Lrp via the linker region.

On the contrary, to activate non-canonical branches the N-terminal part seems to be more important. In contrast to Du et al. [16], who reported that for xWnt5a-driven non-canonical signaling the C-terminus is also involved, our data clearly point to the N-terminal half comprising the BS1 and PS3. As most relevant we identified regions that contribute to the putative oligomerization site PS3 [1], a poorly conserved region in the Wnt proteins. Most likely, recruitment or clustering of different ligand/receptor/co-receptors are involved in the selective activation of distinct non-canonical branches.

Apart from Ror2, which interacts with the Wnt molecule through its CRD domain [22] and therefore through a similar domain as Fz and which is known to be recruited by xWnt5a to clusters [23], little is known about the recruitment of other non-canonical co-receptors. Our data might help to address this in more detail. For Ryk, it has been speculated that the interaction is mediated through the thumb (BS1) and index finger (BS2) of the Wnt-molecule [24]. PTK7 is important for CE movements but does not interact with Wnt5a or Wnt11 [25, 26]. Our characterization of the regions necessary for the activation of different branches of the Wnt-signaling network might help to identify and characterize specific ligand/receptor/co-receptor platforms activating distinct branches of the network. If PS3, as Janda et al. reported [1], indeed induces oligomerization of Wnt/Fz complexes, one can speculate that selective non-canonical pathway activation is not triggered by single Wnt proteins binding to single receptors, but instead depends on the composition of Wnt/Fz oligomers organizing the formation of distinct signaling complexes.

In *Xenopus* gastrulae, the specific response to the Wnt11/PCP and the Wnt5a/Ror2 branches of the non-canonical Wnt-signaling network is the regulation of CE movements [9, 13, 27]. Herein, the distinct branches regulate different aspects of CE movements. While xWnt11 is necessary to reorganize the microtubule cytoskeleton to polarize the cells of the dorsal mesoderm [13], xWnt5a activates a so-called Wnt5a/Ror2 pathway, activating JNK and regulating the expression of the PAPC [9]. However, in general, only little is known about the regulation of non-canonical Wnt target genes. This might be due to the fact that many aspects of the cellular response to non-canonical Wnts relies on restructuring the cytoskeleton rather than on transcriptional regulation [17]. Consistently, in a transcriptome analysis, we found only few genes regulated by xWnt5a and xWnt11. In this assay, we could not confirm that the expression of PAPC in the axial mesoderm is regulated by xWnt5a. Instead, we identified two novel target genes, *pbk* and *rab11fip5*, which are regulated by distinct branches of the non-canonical network. *Pbk* is a target of xWnt5a, *rab11fip5* is a target of xWnt11. Both target genes are involved in the regulation of CE movements. Interestingly, overexpression of the xWnt5a target gene *pbk* phenocopies xWnt5a. The DMZ explants elongate, but fail to constrict. Overexpression of the xWnt11 target gene *rab11fip5* instead phenocopies overexpression of xWnt11. The DMZ explants fail to elongate. Loss of function experiments demonstrate that both target genes, *pbk* and *rab11fip5*, are necessary for CE movements. These data indicate that at least parts of the specific response of the axial mesoderm tissue towards xWnt5a and xWnt11 relies on the expression of their target genes. For *pbk* we could show in epistasis experiments that this novel xWnt5a target gene is the main effector of endogenous xWnt5a in regulating CE movements. Our result that overexpressed xWnt5a did not suppress *pbk* expression indicates that xWnt5a is necessary, but not sufficient to suppress *pbk* expression.

Further analyses have to decipher the molecular mechanisms of how these target genes regulate CE movements. One might speculate that similar to lung cancer cells, *pbk* activates the PI3K/PTEN/AKT signaling pathway through modulation of the protein level of the phosphatase PTEN [28]. Indeed, the regulation of PTEN and the activation of PI3K are important for CE movements in *Xenopus* [29]. Additionally, pbk has been shown to act as MAPKK-like kinase and is highly expressed in various types of cancer such as lymphoma, leukemia, breast cancer, and colorectal cancers [30–34]. During CE movements, the MAPK mediated Erb signaling is important for cell migration. Thus, pbk might act as MAPKK to regulate gastrulation movements [29]. Our analysis of the *Xenopus* DMZ explants links *pbk* for the first time to the Wnt-signaling network. However, whether the migration of the dorsal mesodermal cells towards the midline indeed depends on the kinase activity of pbk remains elusive.

The xWnt11 target gene *rab11fip5* belongs to the group of rab11-family binding proteins (fip). All five groups of rab11fip proteins share a conserved C-terminal rab11-binding domain and interact with the activated GTP-bound form of rab11 [35]. The rab11 small G-proteins (rab11a, rab11b and rab25) are master regulators of the surface expression of receptors and adhesion molecules [36]. Predominantly, rab11 is localized at the recycling endosomes and is involved in the recycling of various receptors to the cell membrane [37–40]. It appears unlikely that components of the xWnt11 signal transduction pathway are among these rab11 regulated proteins because we could not determine any effect of *rab11fip5* on xWnt11-regulated ATF2-luciferase reporter activation. However, in *Xenopus*, rab11 has been shown to be involved in PCP regulated neural tube closure [41]. Since xWnt11 is responsible for the construction of the bipolar cell shape and rab11fip5 phenocopies xWnt11, one might speculate that rab11fip5 is also involved in the polarity formation. Rab11a is one of the regulators of polarized endosome traffic [37]. By interacting with adaptor proteins, rab11 can form complexes with distinct motor proteins, which enable bidirectional transport along microtubule tracks, as well as actin dependent transport [42, 43]. It could be speculated that rab11fip5 is responsible for the traffic of a subset of proteins along the microtubule cytoskeleton to deposit these proteins at the lateral cell ends to establish the bipolar cell shape of the dorsal mesodermal cells. This might be a mechanism for how xWnt11 establishes a polarized microtubule cytoskeleton [13]. However, since in our epistasis experiments loss of rab11fip5 did not compensate for the loss of xWnt11, both proteins use additional independent mechanisms to regulate CE movements.

Apart from all these speculations about how the new non-canonical Wnt target genes might regulate complex cell movements during gastrulation, it remains surprising that two different branches of the non-canonical Wnt network regulate a small subset of target genes in such a highly specific manner. This highly specific response must be triggered by a ligand subtype-specific activation of a distinct Wnt branch and thus in distinct motifs/domains in the Wnt ligand. Our analysis of the chimera, indeed showed that, to regulate rab11fip5 expression, the N-terminal part including PS3-1 and PS3-2 of xWnt11 is required.

Our characterization of the regions necessary for the activation of different branches of the Wnt-signaling network, together with the identification of target genes specifically regulated by distinct branches of the non-canonical Wnt-signaling network might help elucidate the molecular mechanism through which the different Wnts induce their specific response.

## Conclusions

The decision of which branch of the Wnt-signal network becomes activated by a particular member of the Wnt family relies on distinct regions in the proteins. The activation of the canonical Wnt pathway is triggered by the C-terminal part including BS2, whereas the activation of non-canonical parts is triggered mainly by the N-terminal part including BS1 and the PS3 elements. Herein, predominantly PS3-1 and PS3-2 seem to determine the distinction between the xWnt11 and xWnt5a branches. Interestingly, we show here, for the first time, that different branches of the non-canonical Wnt network regulate the expression of distinct target genes. Furthermore, our epistasis experiments revealed that *pbk* is the effector target gene responsible for the xWnt5a-specific response.

## Methods

### Constructs and in vitro mRNA transcription

xWnt5a-pCS2+ [44], xWnt11-pCS2+ [16] and ATF2-Luciferase [18] were as described. In vitro RNA transcription was performed with the mMessage mMachine Kit (Life Technologies GmbH, Germany). The different parts of the chimeric constructs were amplified from xWnt5a_pCS2+ and xWnt11_pCS2+ and fused by PCR. The chimeric constructs were subcloned into pCS2+ via EcoRI and XhoI.

Antisense morpholino oligonucleotides xWnt5aMO [9], xWnt11MO [45], pbkMO, rab11fipfMO, and standard control morpholino were purchased from Gene Tools (Philomath, USA). The open reading frames of xpbk and xrab11fip5 were amplified from gastrula stage cDNA and subcloned into XhoI or EcoRI/XhoI of pCS2+. Both genes displayed one amino acid exchange compared to database sequences (*pbk* NM 001095491.1, *rab11fip5* NM_001091439). For pbk, K258 is substituted by an E; for rab11fip5, S381 is substituted by a F. For the rescue constructs pbkmut and rab11fip5mut silent mutations were introduced by site-directed mutagenesis. Morpholino sequences: pbk 5′-ACTATTCGTGTCCTGCATTTTGGGC-3, rab11fip5: 5′-CGAA GAAACATGAGGACGAGCCTCT-3′.

### *Xenopus* embryos, micromanipulation, DMZ explants in situ hybridization

*Xenopus* embryos were obtained by in vitro fertilization and staged according to Nieuwkoop and Faber [46]. The embryos were injected in dorsal or ventral blastomeres at 4-cell or 8-cell stage and co-injected as lineage tracer Dextran-FITC (Life Technologies GmbH, Germany).

DMZ explants were dissected at stage 10.25 and cultivated in petri dishes coated with 1 % BSA in 1× MBSH until their siblings reached stage 12. The DMZ explants were scored according to three defined phenotypes: (1) elongation and constriction (example: control in Fig. 1a), (2) elongation without constriction (example: xWnt5a in Fig. 1a), and (3) no elongation (example: xWnt11 in Fig. 1a). The classification into the three different categories as shown in Fig. 1 is based on length and width of the protrusion. In wild-type explants (phenotype 1) the protrusion is long and narrow and the length-to-width ratio is > 1.5. In elongated explants that fail to constrict the length and width of the protrusion are more or less equal and/or the protrusion narrows down towards their end (phenotype 2). The length-to-width ratio in these explants ranges between ≈ 0.7 and 1.5. For quantification of the relative elongation, wild-type explants and phenotype 2 explants were counted as elongated explants. When the elongation process is blocked, no tissue protrusion is formed (phenotype 3), and a constriction cannot be observed.

For quantification of the phenotypes we counted the number of embryos showing the indicated phenotypes and calculated the relative elongation (n – nPT3)/n, with n being the total amount of explants and nPT3 the number of explants with phenotype 3. Relative constriction (n – nPT2)/n was only determined for elongated explants, with n being the total amount of elongated explants and nPT2 the number of explants with phenotype 2. In control explants about 10 % of the explants failed to elongate, and about 10 % of the elongated explants failed to constrict. To minimize the influence of different embryo batches we normalized relative elongation and relative constriction in each experiment to control explants of siblings. Significant differences were determined via Fisher’s exact test. The error bars indicate the standard error between N independent experiments (biological replicates), which means that different batches of mRNA were injected in embryos of different parents.

In situ hybridization was performed as described earlier [47]. Antisense Dig-labeled probes were synthesized with the DIG RNA labeling Kit (Roche Applied Science, Germany) using template cDNA encoding xChordin [48]. The embryos were scored according to three defined phenotypes: (1) stretched notochord (*chordin* expression as a narrow stripe, mild phenotype), (2) broadened notochord (*chordin* expression as a broad stripe, mild phenotype) and (3) stuck chordamesoderm (*chordin* expression remained at the blastopore, severe phenotype). The significance level was determined via the χ^2^ test. The error bars indicate the standard error between N independent experiments (biological replicates).

### RNA isolation from DMZ explants, microarray and nanostring analyses

For microarray and nanostring analyses, the total RNA was isolated from 30 DMZ explants at stage 12 via TRIzol Plus RNA Purification kit (Life Technologies GmbH, Germany) according to the manufacturer’s instructions. For the microarray analysis the total RNA was concentrated by RNeasy MinElute Cleanup Kit (Qiagen, Germany) according to the manufacturer’s instructions. The integrity number of total RNA (RIN) was determined via a Bioanalyzer (Agilent Technologies, Germany). For further analyses only samples with a RIN > 8 were used.

The microarray analysis was performed on the *Xenopus* 4 × 44 K gene expression Chip (Agilent Technologies, Germany) by Atlas Biolabs (Berlin, Germany). Data sets are deposited on http://www.ncbi.nlm.nih.gov/geo/query/acc.cgi?acc=GSE81924. Spots displaying more than two-fold difference between morphants and control siblings at a significance level < 0.05 were selected as putative targets.

Nanostring analysis was performed by nCounter (Heidelberg, Germany) [49]. Genes that displayed more than two-fold difference between morphants and control siblings and a significance level < 0.05 according to one-sample *t* test were selected as confirmed target genes.

### TNT and western blotting

TNT coupled reticulocyte lysate system (Promega, Germany) was performed according to manufacturer’s instruction. The biotinylated proteins were detected via an AP conjugated Streptavidin antibody (Promega, Germany) and visualized with NBT/BCIP.

### Transfection and reporter assay

The HEK293 cells were transfected by calcium phosphate precipitation with the reporter ATF2-Luciferase [18], CMV-β-galactosidase and the indicated DNA construct according to Gorman et al. [50]; 48 h after transfection luciferase activity was determined as previously described [51].

## Abbreviations

BS1, binding site 1; BS2, binding site 2; CE, convergent extension; DMZ, dorsal marginal zone; Fz, Frizzled; Pbk, PDZ binding kinase/T-cell originated protein kinase; PS3, pseudosite 3; Rab11fip5, RAB family interacting protein 5

## Additional files

Additional file 1: Figure S1. Overexpressing xWnt5a and xWnt11 disturb convergent extension movements. (A) Overexpression of xWnt5a and (B) xWnt11 results in mislocalization of the *chordin* expression domain ranging from broader expression to an expression that sticks at the blastopore. Quantification of *chordin* phenotypes following (C) xWnt5a and (D) xWnt11 overexpression. Shown is the absolute frequency of the indicated phenotypes. The superimposed error bars illustrate the variation between N independent experiments. N: number of biological replicates, n: number of analyzed embryos, *** *P* < 0.001, according to χ^2^ test, Bars: 500 μm. (TIF 2127 kb) Additional file 2: Figure S5. Analysis of chimera pairs 1 and 2 in DMZ explants. (A) Representative phenotypes of dorsal marginal zone (DMZ) explants of embryos injected with the indicated mRNAs of chimera pair 1. Wnt5a/Wnt11 chimera 1.1 blocks constriction, whereas Wnt11/Wnt5a chimera 1.2 inhibits elongation. (B) Quantification of elongation. Wnt11/Wnt5a chimera 1.2 suppresses elongation in a dose-dependent manner. (C) Quantification of constriction. Wnt5a/Wnt11 chimera 1.1 suppresses constriction in a dose-dependent manner. (D) Representative phenotypes of DMZ explants of embryos injected with the indicated mRNAs of chimera pair 2. Chimera 2.1 and 2.2 influence both elongation and constriction. (E) Quantification of elongation. (F) Quantification of constriction. Shown is the frequency of the indicated phenotypes. In each experiment, the absolute frequency of the indicated phenotypes was normalized to the control siblings. The superimposed error bars illustrate the variation between N independent experiments. N: number of biological replicates, n: number of analyzed explants, *** *P* < 0.001, ** *P* < 0.01, * *P* < 0.05 according to Fisher’s exact test, Bars: 200 μm. (TIF 1428 kb) Additional file 3: Figure S2. The C-terminus determines the activation of the Wnt/β-catenin pathway. (A) Scheme of xWnt8a and xWnt11 constructs fused in a highly conserved region between BS1 and BS2. (B) Ventral injection of xWnt8a and xWnt11/8a resulted in the formation of a secondary axis. xWnt11 and xWnt8a/11 did not induce a secondary axis. (C) Quantification of secondary axis induction. Shown is the absolute frequency of the indicated phenotypes. The superimposed error bars illustrate the variation between N independent experiments. CT: C-terminus; NT: N-terminus; N: number of biological replicates; n: number of analyzed embryos; *** *P* < 0.001 according to Fisher’s exact test; Bars: 500 μm. (TIF 1752 kb) Additional file 4: Figure S3. All chimeras are translated into a protein of the expected size biologically active. In vitro transcribed and translated biotinylated proteins of the chimeric constructs were detected on a western blot via an AP conjugated Streptavidin antibody and visualized with NBT/BCIP. (A) The chimeras between the canonical xWnt8a and the non-canonical xWnt11 are translated in a protein of the expected size. (B) All non-canonical Wnt chimeras are translated in a protein of the expected size. (C) ATF2-luciferase reporter assay of HEK293 cells. All non-canonical chimera pairs are biologically active. Shown is the fold activation of the non-canonical ATF2-luciferase reporter of two independent sets of experiments. The differences in activation between the two sets of experiments are due to different batches of HEK293 cells. In both sets of experiments the chimeric constructs activate the ATF-luciferase reporter in a similar manner as wild-type Wnts. Thus, the chimeras are biologically active non-canonical Wnts. N: number of biological replicates, n: number of independent transfections; * *P* < 0.05, ** *P* < 0.01, *** *P* < 0.001 according to Student’s *t* test. (TIF 676 kb) Additional file 5: Figure S4. xWnt11/8a inhibits elongation. (A) Representative phenotypes of dorsal marginal zone explants of embryos injected with the indicated mRNAs. xWnt8a and xWnt8a/11 do not influence convergent extension movements. The overexpression of xWnt11/8a inhibits elongation. (B) Quantification of elongation. (C) Quantification of constriction. Shown is the frequency of the indicated phenotypes. In each experiment, the absolute frequency of the indicated phenotypes was normalized to the control siblings. The superimposed error bars illustrate the variation between N independent experiments. N: number of biological replicates, n: number of analyzed explants, *** *P* < 0.001, * *P* < 0.05 according to Fisher’s exact test, Bars: 200 μm. (TIF 1236 kb) Additional file 6: Table S1. Putative xWnt5a target gens. Indicating the average fold change (Wnt5a morpholino versus control morpholino) and *P* values of three biological replicates. Targets chosen for further evaluation are highlighted. (DOCX 26 kb) Additional file 7: Table S2. Putative xWnt11 target gens. Indicating the average fold change (Wnt11 morpholino versus control morpholino) and *P* values of three biological replicates. Targets chosen for further evaluation are highlighted. (DOCX 33 kb) Additional file 8: Figure S6. Dynamic expression of pbk and rab11fip5. Maternally expressed *pbk* mRNA (A) is localized at the animal half of the embryo. At gastrula stages no enrichment of *pbk* transcripts at distinct regions is visible. However, half-dissected embryos revealed *pbk* expression mainly in the mesoderm. During the neurula stage, *pbk* mRNA is localized mainly in the developing CNS including the eyes. This localization persists in the tailbud stage. Transversal sections (1 and 2) indicate enriched *pbk* mRNA in the dorsal part of the neural tube (arrows). Until gastrula stages *rab11fip5* expression is similar to *pbk* expression: enriched in the animal half and later concentrated in the mesoderm. From the early neurula stage onward *rab11fip5* is enriched the anterior neuroectoderm (sections 1 and 2). From the late neurula stage onward, an additional ring shaped expression domain is found around the cement gland (section 3, arrow). NT: neural tube, NC: notochord, CG: cement gland. (TIF 10234 kb) Additional file 9: Figure S7. Pbk and rab11fip5 do not interfere with non-canonical Wnt-signaling transduction. (A) Transfected pbk and rab11fip5 do not activate the non-canonical ATF2-luciferase reporter in HEK293 cells. (B) Pbk and rab11fip5 do not interfere with non-canonical ATF2-luciferase reporter activation. N: number of biological replicates, n: number of independent transfections, *** *P* < 0.001, n.s.: not significant according to Student’s *t* test. (TIF 234 kb) Additional file 10: Figure S8. Pbk und rab11fip5Mo. (A) In vitro translated biotinylated proteins of pbk and rab11fip5 were detected on a western blot via an AP conjugated streptavidin antibody and visualized with NBT/BCIP. Addition of antisense morpholino oligonucleotides to the reaction efficiently blocked the production of these proteins. Constructs with silent mutations in the morpholino binding site (pbkmut and rab11fip5mut) are not targeted by the morpholinos. (B) Knock-down of pbk by morpholino-injections in the dorsal equatorial region of four-cell stage embryos resulted in mislocalization of *chordin* expression in a dose-dependent manner. Shown is the frequency of embryos showing mislocalization of chordin expression. The superimposed error bars illustrate the variation between N biological replicates. (C) Some examples of chordin expression in morphants and double injected embryos. n: number of analyzed embryos, *** *P* < 0.001, * *P* < 0.05 according χ^2^ test. (TIF 3910 kb)
