# Supplementary figures and images for: Regulation of distinct branches of the non-canonical Wnt-signaling network in Xenopus dorsal marginal zone explants
Source: BMC Biol. 2016 Jul 5;14:55. doi: 10.1186/s12915-016-0278-x (PMC4932719; doi:10.1186/s12915-016-0278-x)

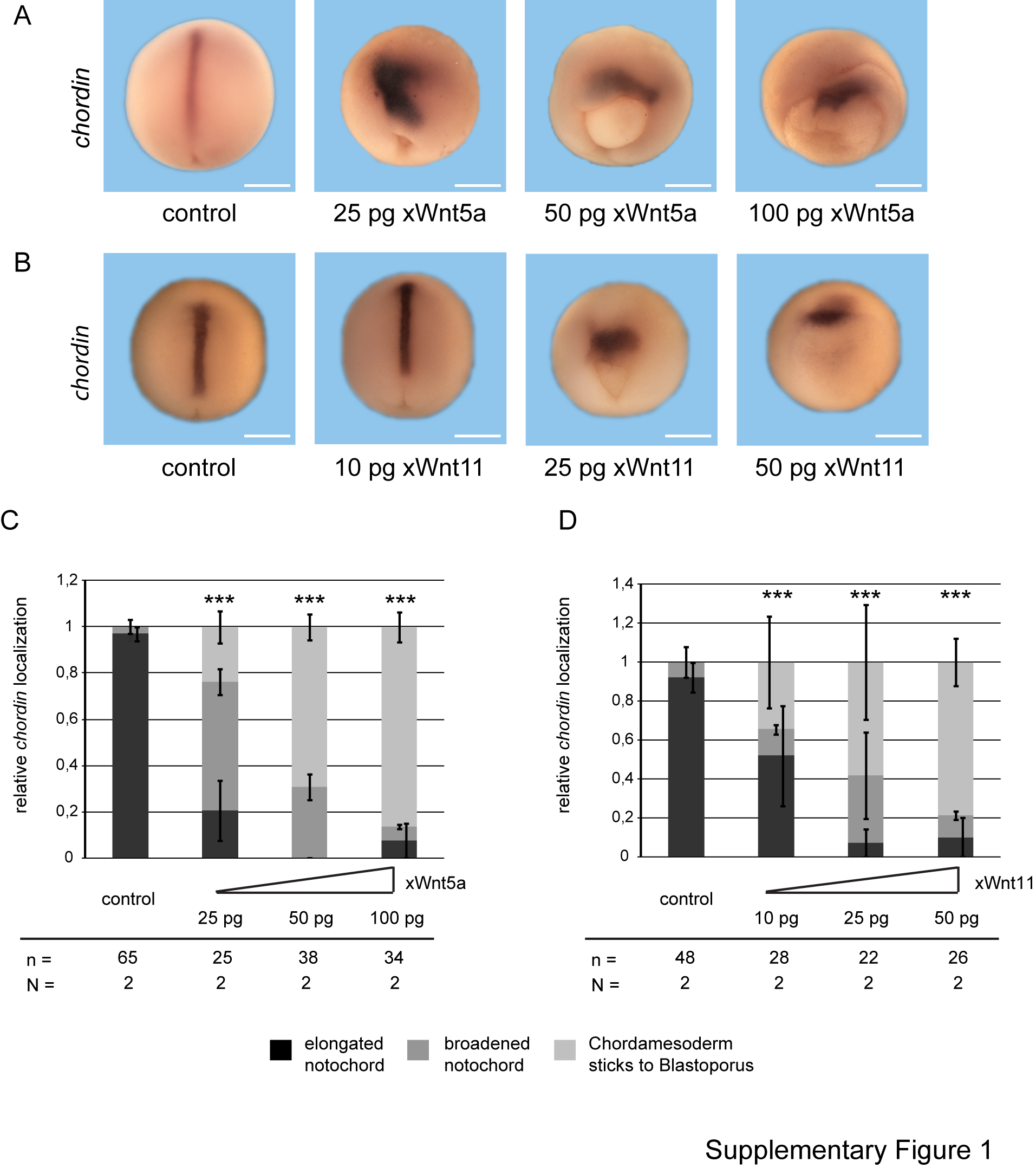

Supplement: Additional file 1: Figure S1. — Overexpressing xWnt5a and xWnt11 disturb convergent extension movements. (A) Overexpression of xWnt5a and (B) xWnt11 results in mislocalization of the chordin expression domain ranging from broader expression to an expression that sticks at the blastopore. Quantification of chordin phenotypes following (C) xWnt5a and (D) xWnt11 overexpression. Shown is the absolute frequency of the indicated phenotypes. The superimposed error bars illustrate the variation between N independent experiments. N: number of biological replicates, n: number of analyzed embryos, *** P < 0.001, according to χ2 test, Bars: 500 μm. (TIF 2127 kb) [file 12915_2016_278_MOESM1_ESM.tif]

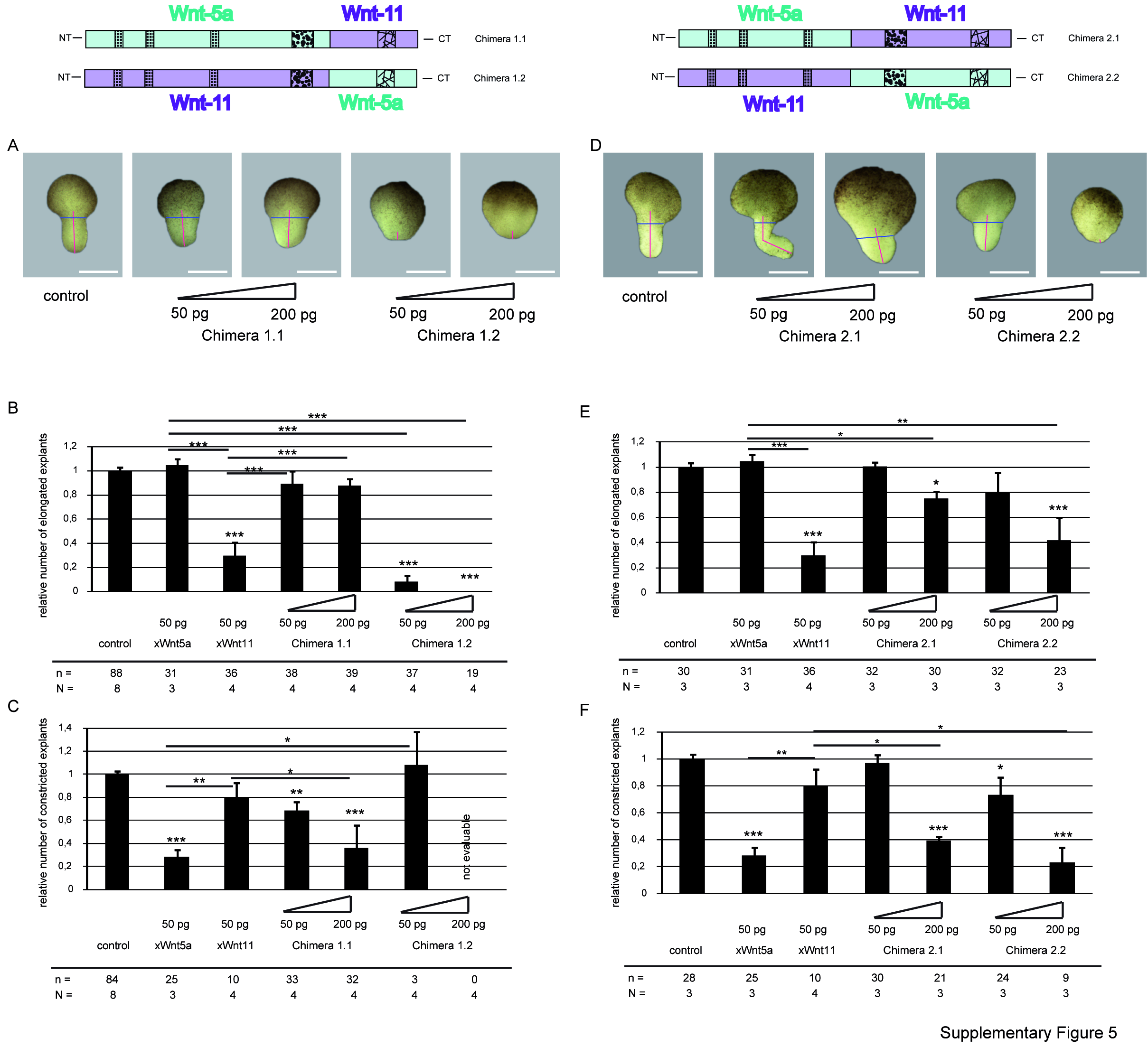

Supplement: Additional file 2: Figure S5. — Analysis of chimera pairs 1 and 2 in DMZ explants. (A) Representative phenotypes of dorsal marginal zone (DMZ) explants of embryos injected with the indicated mRNAs of chimera pair 1. Wnt5a/Wnt11 chimera 1.1 blocks constriction, whereas Wnt11/Wnt5a chimera 1.2 inhibits elongation. (B) Quantification of elongation. Wnt11/Wnt5a chimera 1.2 suppresses elongation in a dose-dependent manner. (C) Quantification of constriction. Wnt5a/Wnt11 chimera 1.1 suppresses constriction in a dose-dependent manner. (D) Representative phenotypes of DMZ explants of embryos injected with the indicated mRNAs of chimera pair 2. Chimera 2.1 and 2.2 influence both elongation and constriction. (E) Quantification of elongation. (F) Quantification of constriction. Shown is the frequency of the indicated phenotypes. In each experiment, the absolute frequency of the indicated phenotypes was normalized to the control siblings. The superimposed error bars illustrate the variation between N independent experiments. N: number of biological replicates, n: number of analyzed explants, *** P < 0.001, ** P < 0.01, * P < 0.05 according to Fisher’s exact test, Bars: 200 μm. (TIF 1428 kb) [file 12915_2016_278_MOESM2_ESM.tif]

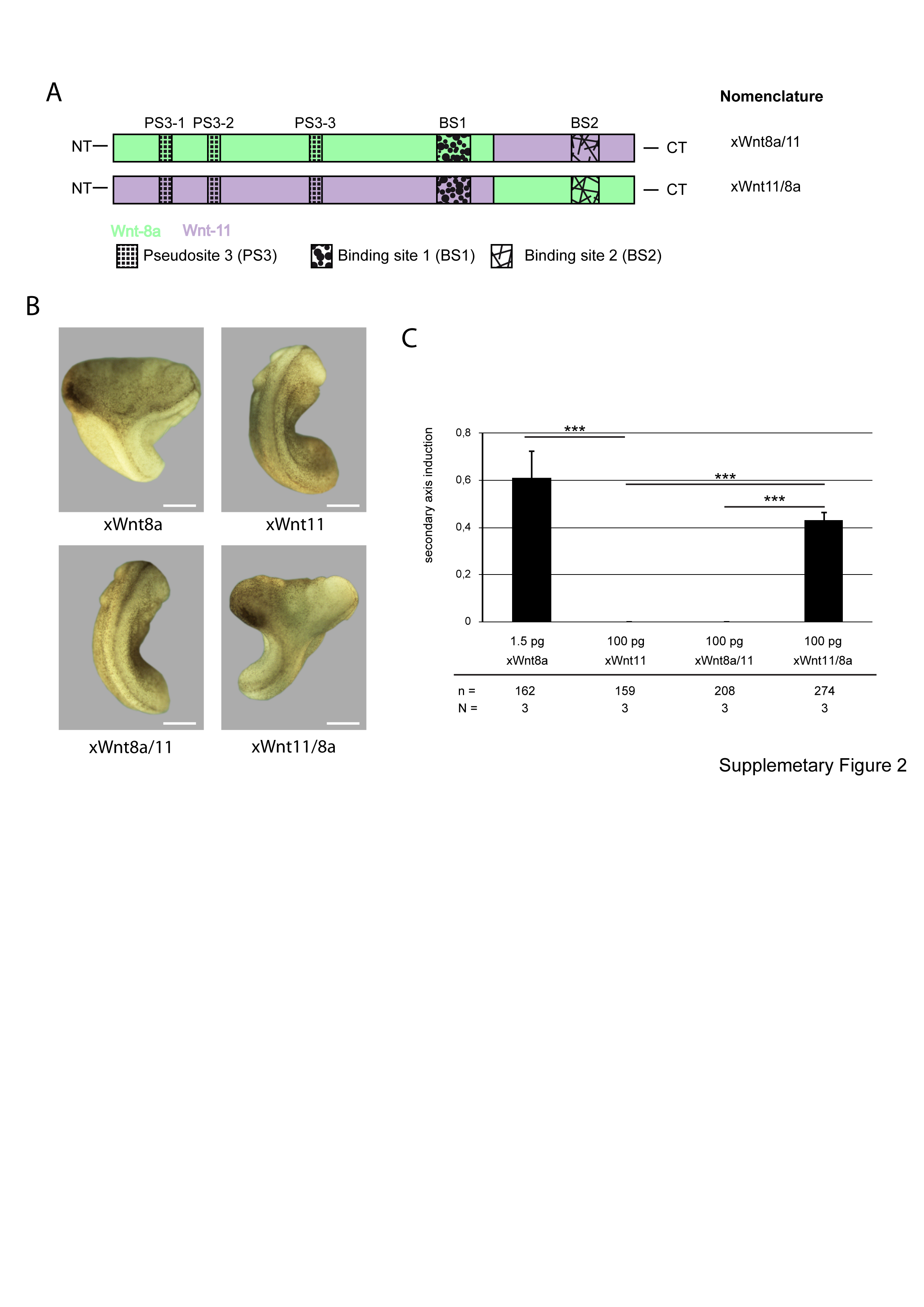

Supplement: Additional file 3: Figure S2. — The C-terminus determines the activation of the Wnt/β-catenin pathway. (A) Scheme of xWnt8a and xWnt11 constructs fused in a highly conserved region between BS1 and BS2. (B) Ventral injection of xWnt8a and xWnt11/8a resulted in the formation of a secondary axis. xWnt11 and xWnt8a/11 did not induce a secondary axis. (C) Quantification of secondary axis induction. Shown is the absolute frequency of the indicated phenotypes. The superimposed error bars illustrate the variation between N independent experiments. CT: C-terminus; NT: N-terminus; N: number of biological replicates; n: number of analyzed embryos; *** P < 0.001 according to Fisher’s exact test; Bars: 500 μm. (TIF 1752 kb) [file 12915_2016_278_MOESM3_ESM.tif]

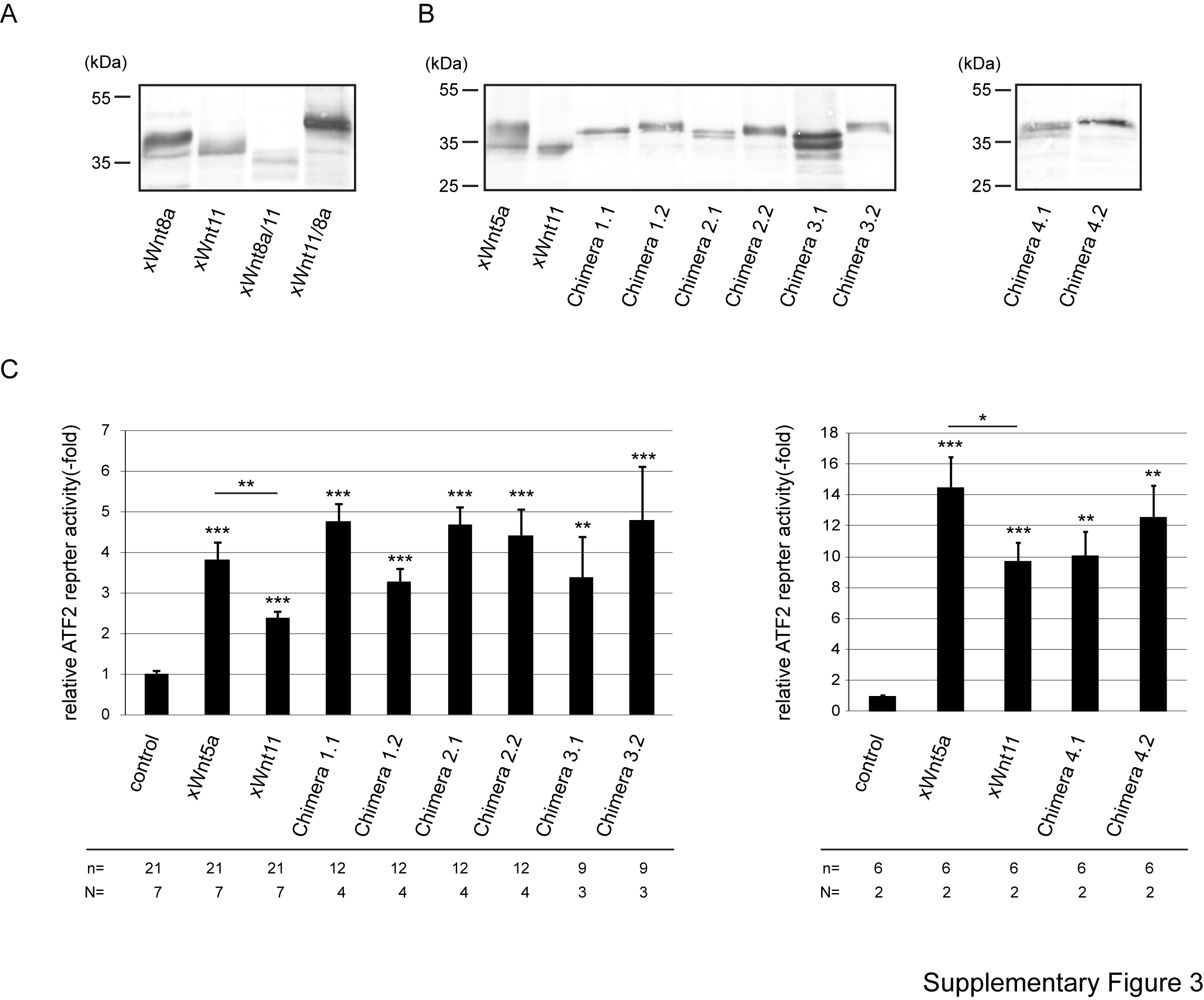

Supplement: Additional file 4: Figure S3. — All chimeras are translated into a protein of the expected size biologically active. In vitro transcribed and translated biotinylated proteins of the chimeric constructs were detected on a western blot via an AP conjugated Streptavidin antibody and visualized with NBT/BCIP. (A) The chimeras between the canonical xWnt8a and the non-canonical xWnt11 are translated in a protein of the expected size. (B) All non-canonical Wnt chimeras are translated in a protein of the expected size. (C) ATF2-luciferase reporter assay of HEK293 cells. All non-canonical chimera pairs are biologically active. Shown is the fold activation of the non-canonical ATF2-luciferase reporter of two independent sets of experiments. The differences in activation between the two sets of experiments are due to different batches of HEK293 cells. In both sets of experiments the chimeric constructs activate the ATF-luciferase reporter in a similar manner as wild-type Wnts. Thus, the chimeras are biologically active non-canonical Wnts. N: number of biological replicates, n: number of independent transfections; * P < 0.05, ** P < 0.01, *** P < 0.001 according to Student’s t test. (TIF 676 kb) [file 12915_2016_278_MOESM4_ESM.tif]

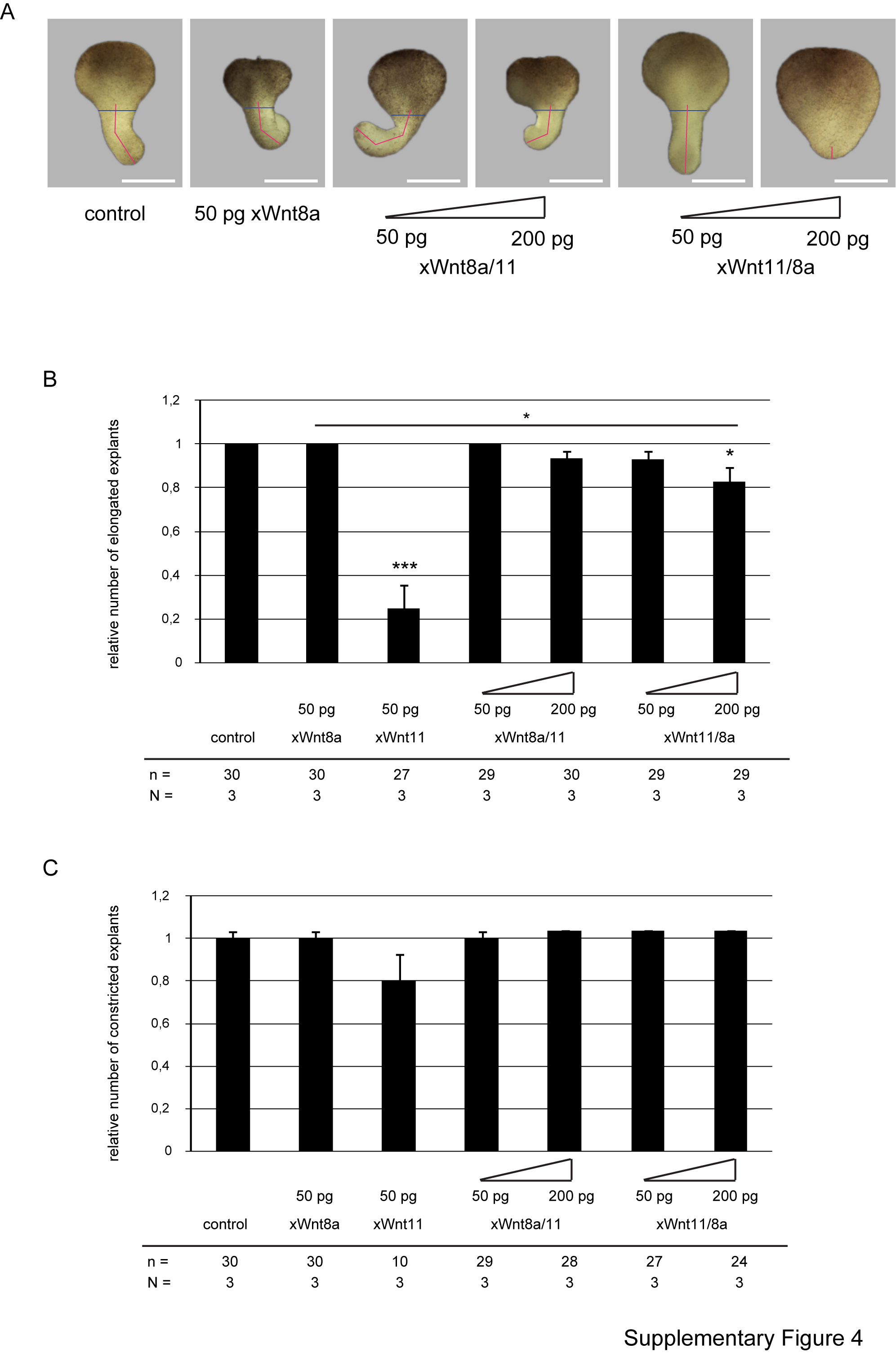

Supplement: Additional file 5: Figure S4. — xWnt11/8a inhibits elongation. (A) Representative phenotypes of dorsal marginal zone explants of embryos injected with the indicated mRNAs. xWnt8a and xWnt8a/11 do not influence convergent extension movements. The overexpression of xWnt11/8a inhibits elongation. (B) Quantification of elongation. (C) Quantification of constriction. Shown is the frequency of the indicated phenotypes. In each experiment, the absolute frequency of the indicated phenotypes was normalized to the control siblings. The superimposed error bars illustrate the variation between N independent experiments. N: number of biological replicates, n: number of analyzed explants, *** P < 0.001, * P < 0.05 according to Fisher’s exact test, Bars: 200 μm. (TIF 1236 kb) [file 12915_2016_278_MOESM5_ESM.tif]

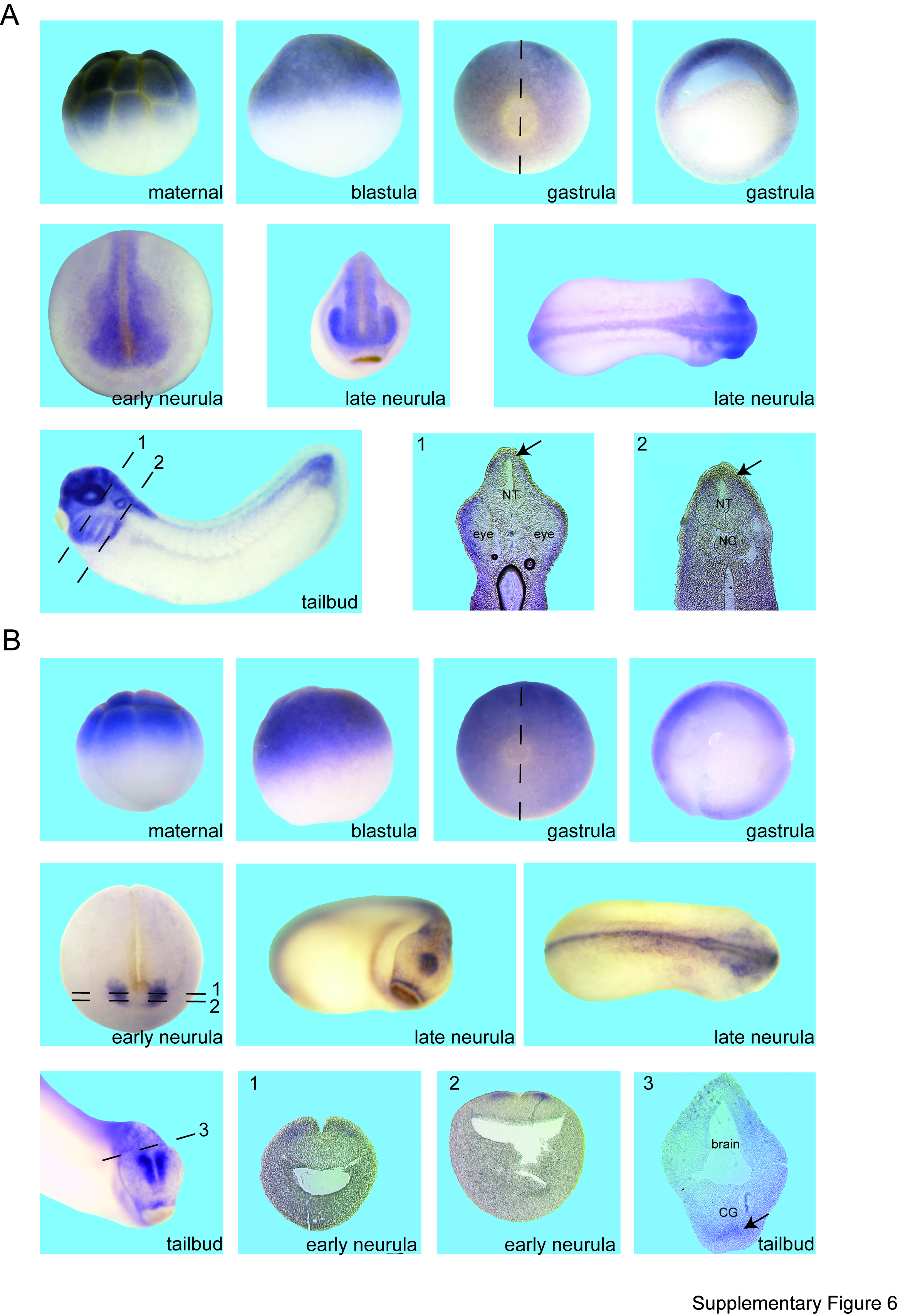

Supplement: Additional file 8: Figure S6. — Dynamic expression of pbk and rab11fip5. Maternally expressed pbk mRNA (A) is localized at the animal half of the embryo. At gastrula stages no enrichment of pbk transcripts at distinct regions is visible. However, half-dissected embryos revealed pbk expression mainly in the mesoderm. During the neurula stage, pbk mRNA is localized mainly in the developing CNS including the eyes. This localization persists in the tailbud stage. Transversal sections (1 and 2) indicate enriched pbk mRNA in the dorsal part of the neural tube (arrows). Until gastrula stages rab11fip5 expression is similar to pbk expression: enriched in the animal half and later concentrated in the mesoderm. From the early neurula stage onward rab11fip5 is enriched the anterior neuroectoderm (sections 1 and 2). From the late neurula stage onward, an additional ring shaped expression domain is found around the cement gland (section 3, arrow). NT: neural tube, NC: notochord, CG: cement gland. (TIF 10234 kb) [file 12915_2016_278_MOESM8_ESM.tif]

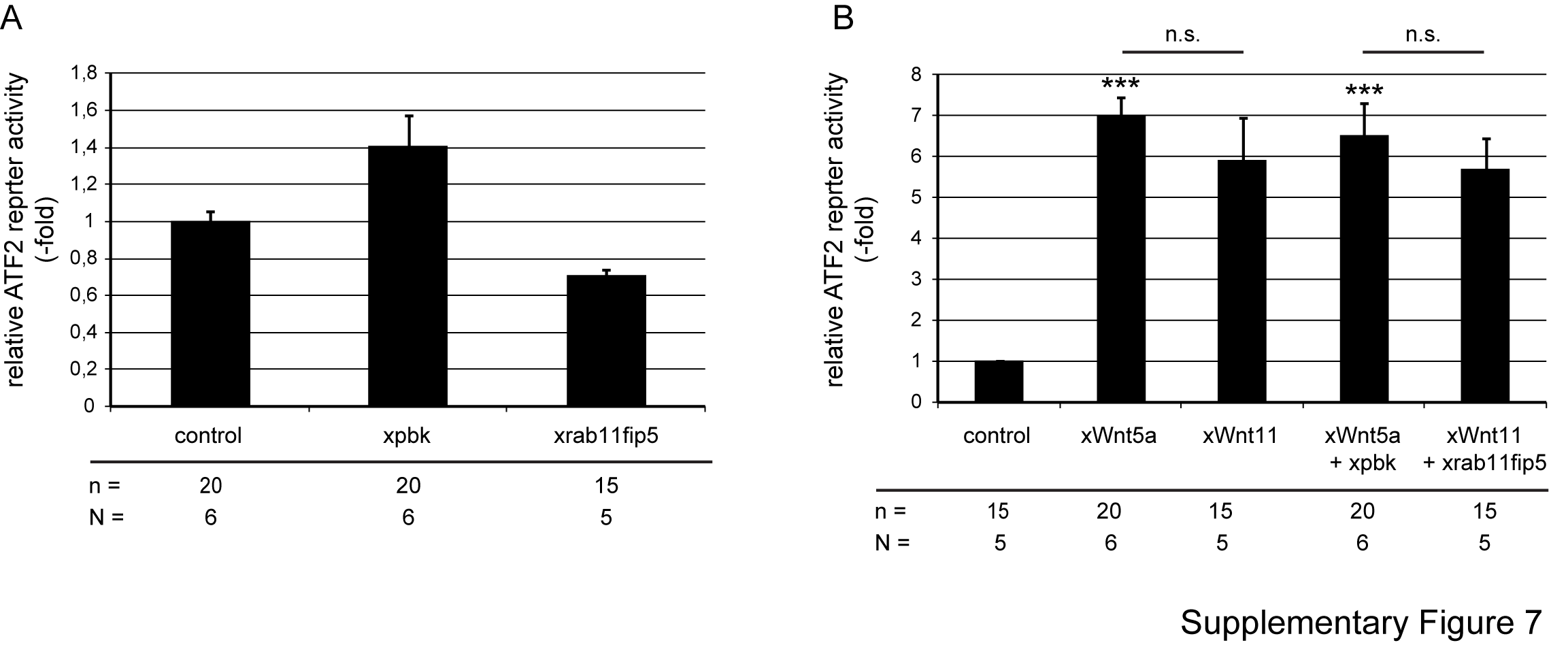

Supplement: Additional file 9: Figure S7. — Pbk and rab11fip5 do not interfere with non-canonical Wnt-signaling transduction. (A) Transfected pbk and rab11fip5 do not activate the non-canonical ATF2-luciferase reporter in HEK293 cells. (B) Pbk and rab11fip5 do not interfere with non-canonical ATF2-luciferase reporter activation. N: number of biological replicates, n: number of independent transfections, *** P < 0.001, n.s.: not significant according to Student’s t test. (TIF 234 kb) [file 12915_2016_278_MOESM9_ESM.tif]

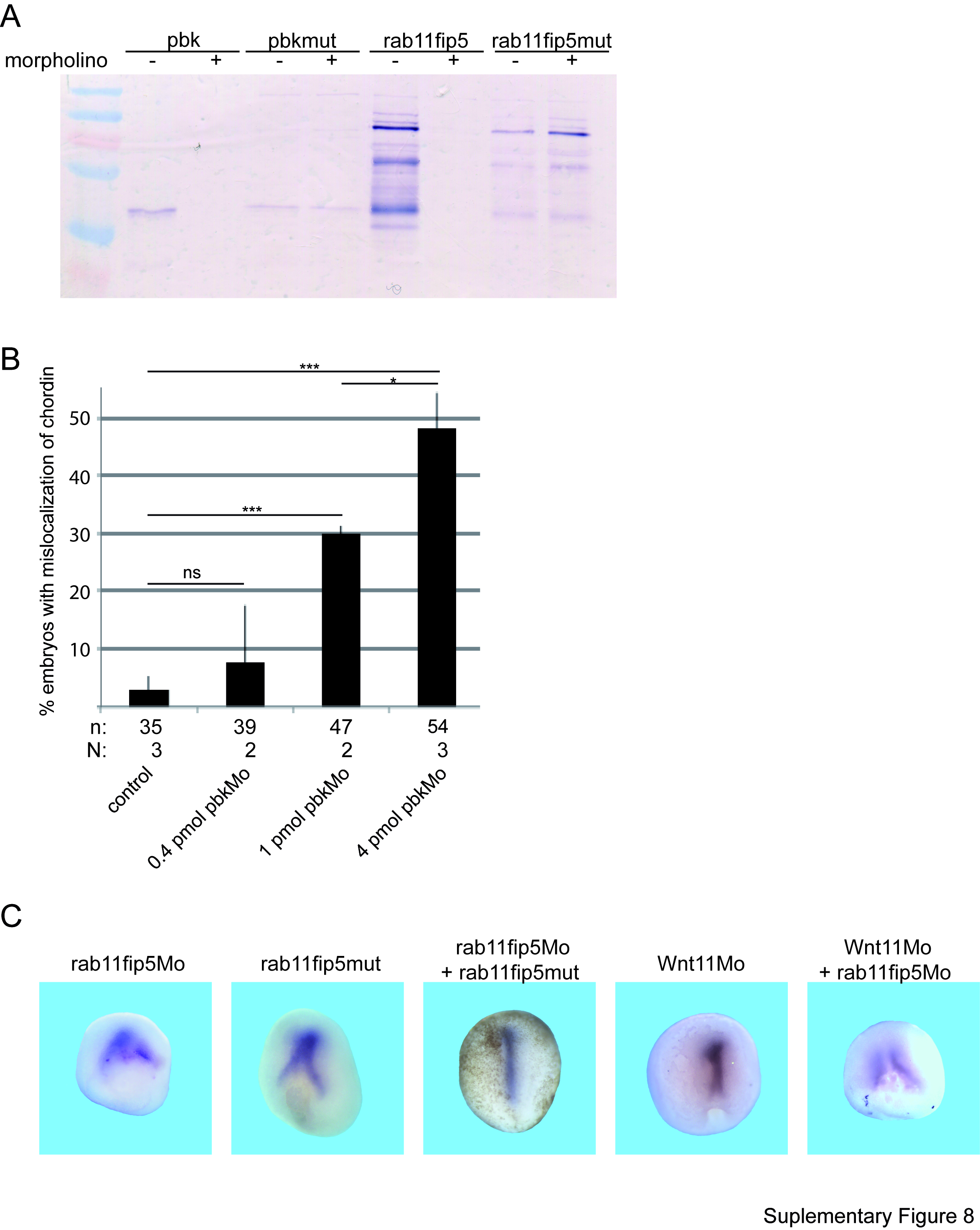

Supplement: Additional file 10: Figure S8. — Pbk und rab11fip5Mo. (A) In vitro translated biotinylated proteins of pbk and rab11fip5 were detected on a western blot via an AP conjugated streptavidin antibody and visualized with NBT/BCIP. Addition of antisense morpholino oligonucleotides to the reaction efficiently blocked the production of these proteins. Constructs with silent mutations in the morpholino binding site (pbkmut and rab11fip5mut) are not targeted by the morpholinos. (B) Knock-down of pbk by morpholino-injections in the dorsal equatorial region of four-cell stage embryos resulted in mislocalization of chordin expression in a dose-dependent manner. Shown is the frequency of embryos showing mislocalization of chordin expression. The superimposed error bars illustrate the variation between N biological replicates. (C) Some examples of chordin expression in morphants and double injected embryos. n: number of analyzed embryos, *** P < 0.001, * P < 0.05 according χ2 test. (TIF 3910 kb) [file 12915_2016_278_MOESM10_ESM.tif]
