# Supplementary material for: Regulation of distinct branches of the non-canonical Wnt-signaling network in Xenopus dorsal marginal zone explants
Source: BMC Biol. 2016 Jul 5;14:55. doi: 10.1186/s12915-016-0278-x (PMC4932719; doi:10.1186/s12915-016-0278-x)
Supplement: Additional file 6: Table S1. — Putative xWnt5a target gens. Indicating the average fold change (Wnt5a morpholino versus control morpholino) and P values of three biological replicates. Targets chosen for further evaluation are highlighted. (DOCX 26 kb) [file 12915_2016_278_MOESM6_ESM.docx]

| 'Probe Id' | 'p-Value' | 'fold_change' | 'Gene_Description' |
| --- | --- | --- | --- |
| 'A_10_P114256' | 0,016814745 | -9,728167256 | 'Unknown' |
| 'A_10_P169253' | 0,008015396 | 7,486368835 | Unknown' |
| 'A_10_P011927' | 0,015894807 | -6,496584747 | 'gb\|daa33d10.y1 NICHD XGC Lu1 Xenopus laevis cDNA clone IMAGE:4058154 5, mRNA sequence [BG233195]' |
| 'A_10_P274067' | 0,011799686 | -5,415850782 | 'Unknown' |
| 'A_10_P048056' | 0,024829042 | -5,320947031 | 'ref\|Xenopus laevis T-cell immunoglobulin and mucin domain containing 4 (timd4), mRNA [NM_001094362]' |
| 'A_10_P076860' | 0,032051215 | 5,167949177 | 'gb\|AGENCOURT_19148001 NICHD_XGC_Te2 Xenopus laevis cDNA clone IMAGE:7211277 5, mRNA sequence [CK805110]' |
| 'A_10_P014039' | 0,012819211 | -5,043069109 | 'gb\|BJ071063 NIBB Mochii normalized Xenopus tailbud library Xenopus laevis cDNA clone XL092n22 5, mRNA sequence [BJ071063]' |
| 'A_10_P022708' | 0,039200023 | -4,272912784 | 'gb\|AGENCOURT_13324664 NICHD_XGC_Tad1 Xenopus laevis cDNA clone IMAGE:6879027 3, mRNA sequence [CB562544]' |
| 'A_10_P072355' | 0,037968142 | -4,205306989 | 'Unknown' |
| 'A_10_P007843' | 0,025105011 | -4,199027256 | 'ref\|Xenopus laevis RNA binding protein with multiple splicing 2 (rbpms2), mRNA [NM_001094266]' |
| A_10_P155823' | 0,008830932 | -4,107508795 | ref\|Xenopus laevis PDZ binding kinase (pbk), mRNA [NM_001095491]' |
| 'A_10_P009349' | 0,00771469 | -3,941715489 | 'ref\|Xenopus laevis PDZ binding kinase (pbk), mRNA [NM_001095491]' |
| 'A_10_P054489' | 0,002134237 | 3,465458742 | 'gb\|za41d01.x1 Xenopus EST library Xenopus laevis cDNA clone za41d01 5, mRNA sequence [AW158429]' |
| 'A_10_P063189' | 0,013072051 | 3,395018944 | 'gb\|AGENCOURT_14150232 NICHD_XGC_Eye1 Xenopus laevis cDNA clone IMAGE:6948313 5, mRNA sequence [CD326328]' |
| 'A_10_P037746' | 0,03495352 | 3,160418855 | 'ref\|Xenopus laevis adenosine deaminase domain containing 1 (testis-specific) (adad1), mRNA [NM_001096430]' |
| 'A_10_P016444' | 0,0485724 | 3,158980761 | 'gb\|BP693645 Osada Taira anterior neuroectoderm (ANE) pCS105 cDNA library Xenopus laevis cDNA clone XL471c17ex 5, mRNA sequence [BP693645]' |
| 'A_10_P124274' | 0,009019946 | 3,154929154 | 'Unknown' |
| A_10_P142528' | 0,026809813 | -3,145244174 | ref\|Xenopus laevis speckle-type POZ protein (spop-b), mRNA [NM_001097009]' |
| A_10_P037821' | 0,013578166 | 3,055979262 | ref\|Xenopus laevis family with sequence similarity 89, member A (fam89a), mRNA [NM_001127825]' |
| 'A_10_P042981' | 0,028754937 | -3,049060593 | ref\|Xenopus laevis speckle-type POZ protein (spop-b), mRNA [NM_001097009]' |
| 'A_10_P256602' | 0,027420651 | -3,021321506 | 'tc\|Rep: Topoisomerase IV subunit B - Streptococcus pneumoniae, partial (12percent) [TC463397]' |
| 'A_10_P004014' | 0,028099324 | -2,953387358 | 'ref\|Xenopus laevis solute carrier family 25 (mitochondrial carrier: glutamate), member 22 (slc25a22), nuclear gene encoding mitochondrial protein, mRNA [NM_001090205]' |
| 'A_10_P164233' | 0,044668375 | 2,835480341 | 'tc\|Rep: Cytochrome c oxidase subunit 2 - Ichthyophis bannanicus (Banna caecilian), partial (6percent) [TC431670]' |
| A_10_P003454' | 0,00983075 | 2,826450426 | ref\|Xenopus laevis shisa 3 (shisa3), mRNA [NM_001086364]' |
| 'A_10_P244058' | 0,016827997 | -2,787246571 | 'ref\|Xenopus laevis acyl-CoA dehydrogenase, long chain (acadl), nuclear gene encoding mitochondrial protein, mRNA [NM_001093365]' |
| 'A_10_P011920' | 0,008372194 | -2,78436739 | 'ref\|Xenopus laevis potassium voltage-gated channel, Isk-related family, member 3 (kcne3), mRNA [NM_001088877]' |
| 'A_10_P126789' | 0,047459794 | -2,715302557 | 'Unknown' |
| 'A_10_P169778' | 0,015562425 | -2,700517762 | 'ref\|Xenopus laevis PDZ binding kinase (pbk), mRNA [NM_001095491]' |
| 'A_10_P125644' | 0,04892536 | -2,684248569 | 'gb\|AGENCOURT_13319100 NICHD_XGC_Tad2 Xenopus laevis cDNA clone IMAGE:6873505 3, mRNA sequence [CB565302]' |
| 'A_10_P211578' | 0,001720285 | -2,679922871 | 'tc\|Rep: Ela3b-prov protein - Xenopus laevis (African clawed frog), partial (45percent) [TC449102]' |
| 'A_10_P112176' | 0,013768763 | 2,608287166 | 'Unknown' |
| 'A_10_P007200' | 0,043784954 | -2,598125887 | 'gb\|Xenopus laevis cDNA clone MGC:82763 IMAGE:5156829, complete cds [BC078036]' |
| 'A_10_P217123' | 0,00931537 | -2,59314196 | 'tc\|Rep: histone cluster 4, H4 - Mus musculus, partial (93percent) [TC451123]' |
| 'A_10_P012969' | 0,00787374 | 2,592788272 | 'gb\|dah97b09.x1 NICHD XGC Emb4 Xenopus laevis cDNA clone IMAGE:4957769 3, mRNA sequence [BI315351]' |
| 'A_10_P190723' | 0,016838107 | 2,580754848 | 'Unknown' |
| 'A_10_P026431' | 0,044514469 | -2,501022059 | 'gb\|AGENCOURT_26179910 Blumberg_Cho Xenopus laevis cDNA clone IMAGE:7299413 5, mRNA sequence [CO387113]' |
| 'A_10_P156693' | 0,030444035 | 2,491388781 | 'tc\|Rep: Cell division ATP-binding protein FtsE precursor - Thermosinus carboxydivorans Nor1, partial (10percent) [TC428693]' |
| 'A_10_P245688' | 0,003171905 | -2,466364552 | 'ref\|Xenopus laevis uncharacterized LOC496152 (LOC496152), mRNA [NM_001095374]' |
| 'A_10_P183808' | 0,008479657 | 2,44727871 | 'Unknown' |
| 'A_10_P264212' | 0,005310842 | -2,438913219 | 'tc\|Rep: Lin-7 homolog B - Homo sapiens (Human), partial (57percent) [TC465537]' |
| 'A_10_P094299' | 0,018621933 | -2,431489396 | 'gb\|DC122901 Yamamoto [DC122901]' |
| 'A_10_P006820' | 0,040391742 | -2,398632027 | 'ref\|Xenopus laevis acyl-CoA dehydrogenase, long chain (acadl), nuclear gene encoding mitochondrial protein, mRNA [NM_001093365]' |
| 'A_10_P002029' | 0,020897678 | -2,388741838 | 'ref\|Xenopus laevis zygote arrest 1 (zar1), mRNA [NM_001090489]' |
| 'A_10_P056862' | 0,015288042 | 2,370853133 | 'gb\|BJ046737 NIBB Mochii normalized Xenopus neurula library Xenopus laevis cDNA clone XL014f07 3, mRNA sequence [BJ046737]' |
| 'A_10_P107421' | 0,00361185 | 2,359025722 | 'gb\|AGENCOURT_14218806 NICHD_XGC_Eye1 Xenopus laevis cDNA clone IMAGE:6946280 5, mRNA sequence [CD361615]' |
| 'A_10_P026829' | 0,006548866 | -2,354877704 | 'gb\|AGENCOURT_39734825 NICHD_XGC_Te2N Xenopus laevis cDNA clone IMAGE:7764957 3, mRNA sequence [CX133078]' |
| 'A_10_P016709' | 0,037073707 | 2,34249468 | 'gb\|BP705198 Osada Taira anterior neuroectoderm (ANE) pCS105 cDNA library Xenopus laevis cDNA clone XL514c23ex 5, mRNA sequence [BP705198]' |
| 'A_10_P058837' | 0,000695935 | -2,323123241 | 'gb\|Xenopus laevis cDNA clone IMAGE:6865205 [BC127426]' |
| 'A_10_P064354' | 0,037399758 | -2,316607922 | 'gb\|AGENCOURT_39735527 NICHD_XGC_Te2N Xenopus laevis cDNA clone IMAGE:7765649 3, mRNA sequence [CX132668]' |
| 'A_10_P076835' | 0,01105658 | -2,295972169 | 'gb\|AGENCOURT_10125350 NICHD_XGC_Kid1 Xenopus laevis cDNA clone IMAGE:4031190 5, mRNA sequence [BU903735]' |
| 'A_10_P017098' | 0,027258167 | 2,276309919 | 'gb\|BP726258 Osada Taira anterior neuroectoderm (ANE) pCS105 cDNA library Xenopus laevis cDNA clone XL466k14ex 3, mRNA sequence [BP726258]' |
| 'A_10_P250052' | 0,010529425 | -2,260602039 | 'Unknown' |
| 'A_10_P155993' | 0,022814042 | 2,225184277 | 'ref\|Xenopus laevis uncharacterized protein MGC115716 (MGC115716), mRNA [NM_001096096]' |
| 'A_10_P140863' | 0,012895513 | 2,208989711 | 'tc\|Rep: Glutamine synthetase - Xenopus tropicalis (Western clawed frog) (Silurana tropicalis), complete [TC417586]' |
| 'A_10_P251242' | 0,043283468 | 2,201999247 | 'gb\|AGENCOURT_11113799 NICHD XGC Emb1 Xenopus laevis cDNA clone IMAGE:6863661 5, mRNA sequence [CA987095]' |
| 'A_10_P134448' | 0,030098631 | -2,191465591 | 'Unknown' |
| 'A_10_P208418' | 0,002286066 | 2,186720107 | 'tc\|Rep: EF hand family protein - Tetrahymena thermophila SB210, partial (8percent) [TC447950]' |
| 'A_10_P183353' | 0,022180948 | 2,169200784 | tc\|Rep: Elk3-prov protein - Xenopus laevis (African clawed frog), partial (52percent) [TC438870]' |
| 'A_10_P016288' | 0,00180187 | 2,166761195 | 'gb\|BP687326 Osada Taira anterior neuroectoderm (ANE) pCS105 cDNA library Xenopus laevis cDNA clone XL453o10ex 5, mRNA sequence [BP687326]' |
| 'A_10_P022599' | 0,010137197 | 2,124828823 | 'gb\|AGENCOURT_13329763 NICHD_XGC_Tad1 Xenopus laevis cDNA clone IMAGE:6880045 3, mRNA sequence [CB561564]' |
| 'A_10_P006614' | 0,027289484 | 2,123081436 | 'ref\|Xenopus laevis TP53RK binding protein (tprkb), mRNA [NM_001093209]' |
| 'A_10_P105870' | 0,025952772 | -2,100501141 | 'gb\|AGENCOURT_8098790 NICHD XGC Emb4 Xenopus laevis cDNA clone IMAGE:5570849 5, mRNA sequence [BQ735822]' |
| 'A_10_P022928' | 0,020990574 | -2,064692322 | 'gb\|AGENCOURT_13325678 NICHD_XGC_Tad2 Xenopus laevis cDNA clone IMAGE:6872804 3, mRNA sequence [CB564821]' |
| 'A_10_P058987' | 0,037591695 | -2,06366475 | 'gb\|AGENCOURT_26186102 Blumberg_Cho Xenopus laevis cDNA clone IMAGE:7297087 5, mRNA sequence [CO384583]' |
| 'A_10_P219663' | 0,027386897 | -2,037221522 | 'Unknown' |
| 'A_10_P081860' | 0,026052528 | -2,02340956 | 'gb\|da74b05.x1 Harland stage 19-23 Xenopus laevis cDNA clone IMAGE:3200625 3, mRNA sequence [AW766346]' |
| 'A_10_P264547' | 0,03469949 | -2,017123243 | 'tc\|Rep: LigA - Methylobacterium sp. 4-46, partial (6percent) [TC465623]' |

## Supplementary table 1: Putative xWnt5a target genes
