# Supplementary material for: Regulation of distinct branches of the non-canonical Wnt-signaling network in Xenopus dorsal marginal zone explants
Source: BMC Biol. 2016 Jul 5;14:55. doi: 10.1186/s12915-016-0278-x (PMC4932719; doi:10.1186/s12915-016-0278-x)
Supplement: Additional file 7: Table S2. — Putative xWnt11 target gens. Indicating the average fold change (Wnt11 morpholino versus control morpholino) and P values of three biological replicates. Targets chosen for further evaluation are highlighted. (DOCX 33 kb) [file 12915_2016_278_MOESM7_ESM.docx]

| 'Probe Id' | 'p-Value' | 'fold_change' | 'Gene_Description' |
| --- | --- | --- | --- |
| 'A_10_P169253' | 0,003293172 | 7,317693898 | 'Unknown' |
| 'A_10_P067627' | 0,041477194 | -7,143187631 | 'gb\|AGENCOURT_11210731 NICHD_XGC_Tad2 Xenopus laevis cDNA clone IMAGE:6873955 5, mRNA sequence [CB198950]' |
| 'A_10_P125429' | 0,046354483 | -6,538453212 | 'Unknown' |
| 'A_10_P009076' | 0,003036147 | 5,861016625 | ref\|Xenopus laevis folliculogenesis specific basic helix-loop-helix (figla), mRNA [NM_001095198]' |
| 'A_10_P017235' | 0,013897359 | -5,684996231 | 'gb\|BP735133 Osada Taira anterior neuroectoderm (ANE) pCS105 cDNA library Xenopus laevis cDNA clone XL504d05ex 3, mRNA sequence [BP735133]' |
| 'A_10_P001187' | 0,009737473 | -5,350420283 | 'tc\|Rep: X.laevis nervous system-specific RNA-binding protein - Xenopus laevis (African clawed frog), partial (15percent) [TC455215]' |
| 'A_10_P076860' | 0,02725891 | 4,948483403 | 'gb\|AGENCOURT_19148001 NICHD_XGC_Te2 Xenopus laevis cDNA clone IMAGE:7211277 5, mRNA sequence [CK805110]' |
| 'A_10_P127259' | 0,029309925 | -4,788506142 | 'Unknown' |
| 'A_10_P046841' | 0,020636499 | -4,775043777 | 'ref\|Xenopus laevis uncharacterized LOC100101281 (LOC100101281), mRNA [NM_001099872]' |
| 'A_10_P140863' | 0,008296086 | 4,768321485 | 'tc\|Rep: Glutamine synthetase - Xenopus tropicalis (Western clawed frog) (Silurana tropicalis), complete [TC417586]' |
| 'A_10_P020549' | 0,01753659 | 4,280661549 | 'gb\|BX852510 NICHD_XGC_Lu1 Xenopus laevis cDNA clone IMAGp998E029326 ; IMAGE:4056937 5, mRNA sequence [BX852510]' |
| 'A_10_P050869' | 0,020769012 | -4,017881846 | 'gb\|AGENCOURT_13042454 NICHD_XGC_Te1 Xenopus laevis cDNA clone IMAGE:6897772 5, mRNA sequence [CB592108]' |
| 'A_10_P213063' | 0,000639801 | -3,946394518 | 'tc\|Rep: Integrase - Streptomyces phage TG1, partial (4percent) [TC449684]' |
| 'A_10_P260832' | 0,018210608 | -3,92280959 | 'Unknown' |
| 'A_10_P073520' | 0,028819454 | -3,856203034 | 'gb\|AGENCOURT_13045150 NICHD_XGC_Te1 Xenopus laevis cDNA clone IMAGE:6897025 5, mRNA sequence [CB593336]' |
| 'A_10_P020720' | 0,00832637 | -3,849600578 | 'gb\|BX854265 NICHD_XGC_Emb3 Xenopus laevis cDNA clone IMAGp998E038299 ; IMAGE:3400682 5, mRNA sequence [BX854265]' |
| 'A_10_P276607' | 0,039765966 | -3,703328416 | 'tc\|Rep: High mobility group protein 20A - Xenopus laevis (African clawed frog), partial (46percent) [TC468842]' |
| 'A_10_P215778' | 0,04058423 | -3,564614755 | 'tc\|Rep: Predicted protein - Nematostella vectensis (Starlet sea anemone), partial (6percent) [TC450655]' |
| 'A_10_P016606' | 0,029854299 | -3,446461001 | 'gb\|BP701172 Osada Taira anterior neuroectoderm (ANE) pCS105 cDNA library Xenopus laevis cDNA clone XL500k14ex 5, mRNA sequence [BP701172]' |
| 'A_10_P010235' | 0,005834556 | -3,4443528 | 'tc\|Rep: ABC-type transport system involved in resistance to organic solvents, auxiliary component - Lawsonia intracellularis (strain PHE/MN1-00), partial (11percent) [TC447233]' |
| 'A_10_P047761' | 0,007762153 | 3,434984885 | ref\|Xenopus laevis tubulin, gamma 1 (tubg1), mRNA [NM_001090573]' |
| 'A_10_P137148' | 0,039581405 | -3,345499724 | 'Unknown' |
| 'A_10_P089645' | 0,011169237 | -3,255960939 | 'gb\|NISC_mo02b08.x1 Soares NXEG Xenopus laevis cDNA clone IMAGE:5162055 3, mRNA sequence [BQ397804]' |
| 'A_10_P019797' | 0,041608322 | -3,248689157 | 'gb\|BX844434 Wellcome CRC pSK egg Xenopus laevis cDNA clone IMAGp998K188240 ; IMAGE:3378185 5, mRNA sequence [BX844434]' |
| 'A_10_P168718' | 0,006544334 | 3,173708669 | 'Unknown' |
| 'A_10_P146058' | 0,027440462 | -3,160584709 | ref\|Xenopus laevis teratocarcinoma-derived growth factor 1, member 1 (tdgf1.1), mRNA [NM_001095666]' |
| 'A_10_P027234' | 0,031766625 | -3,129835425 | ref\|Xenopus laevis HESX homeobox 1 (hesx1-a), mRNA [NM_001085795]' |
| 'A_10_P002433' | 0,031047572 | 3,122304161 | ref\|Xenopus laevis similar to tektin 2 (testicular) (MGC52862), mRNA [NM_001085998]' |
| 'A_10_P048061' | 0,004323137 | -3,039974925 | 'ref\|Xenopus laevis telomerase RNA (terc), telomerase RNA [NR_003556]' |
| 'A_10_P182038' | 0,035489202 | -3,039180363 | 'Unknown' |
| 'A_10_P245688' | 0,005968442 | -3,007865525 | 'ref\|Xenopus laevis uncharacterized LOC496152 (LOC496152), mRNA [NM_001095374]' |
| 'A_10_P069237' | 0,042592688 | 2,996071422 | 'gb\|Xenopus laevis hypothetical protein LOC733204, mRNA (cDNA clone IMAGE:7019135), partial cds [BC093579]' |
| 'A_10_P083800' | 0,009489127 | -2,978798709 | 'gb\|AGENCOURT_69679494 NICHD_XGC_Ov1 Xenopus laevis cDNA clone IMAGE:8329599 5, mRNA sequence [DY556442]' |
| 'A_10_P024441' | 0,017948635 | -2,968676603 | 'gb\|AGENCOURT_14147284 NICHD_XGC_Brn1 Xenopus laevis cDNA clone IMAGE:6950474 3, mRNA sequence [CD329691]' |
| 'A_10_P170223' | 0,004708274 | -2,876301635 | ref\|Xenopus laevis RNA binding motif protein 26 (rbm26), mRNA [NM_001085999]' |
| 'A_10_P013499' | 0,026440671 | 2,85172763 | 'gb\|BJ046006 NIBB Mochii normalized Xenopus neurula library Xenopus laevis cDNA clone XL005p05 3, mRNA sequence [BJ046006]' |
| 'A_10_P058362' | 0,026871179 | -2,828088918 | 'gb\|AGENCOURT_77792935 NICHD_XGC_skin_m Xenopus laevis cDNA clone IMAGE:8643564 5, mRNA sequence [EB729160]' |
| 'A_10_P026506' | 0,006167897 | 2,820642432 | 'gb\|AGENCOURT_26180146 Blumberg_Cho Xenopus laevis cDNA clone IMAGE:7299691 5, mRNA sequence [CO389756]' |
| 'A_10_P049889' | 0,019701685 | -2,817506623 | 'Unknown' |
| 'A_10_P105895' | 0,022958115 | 2,799543386 | 'gb\|AGENCOURT_15189709 NICHD_XGC_Sp1 Xenopus laevis cDNA clone IMAGE:5512955 5, mRNA sequence [CF270358]' |
| 'A_10_P253112' | 0,038079821 | -2,799234176 | tc\|Rep: Multiple myeloma tumor-associated protein 2 (hMMTAG2). - Xenopus tropicalis, partial (9percent) [TC462403]' |
| 'A_10_P105450' | 0,006674219 | 2,781251812 | gb\|AGENCOURT_14149597 NICHD_XGC_Eye1 Xenopus laevis cDNA clone IMAGE:6947950 5, mRNA sequence [CD326939]' |
| 'A_10_P017845' | 0,022461538 | -2,716453354 | gb\|AGENCOURT_8216698 NICHD XGC Emb4 Xenopus laevis cDNA clone IMAGE:4930327 5, mRNA sequence [BQ731458]' |
| 'A_10_P013420' | 0,009199946 | -2,711492702 | 'Unknown' |
| 'A_10_P224633' | 0,040348959 | -2,700324905 | 'tc\|Rep: Trypsin precursor - Xenopus laevis (African clawed frog), partial (59percent) [TC453650]' |
| 'A_10_P100610' | 0,033649871 | -2,697330243 | 'gb\|Xenopus laevis cDNA clone IMAGE:6632310 [BC128983]' |
| 'A_10_P122704' | 0,033137377 | -2,656589913 | 'gb\|AGENCOURT_13045736 NICHD_XGC_Kid1 Xenopus laevis cDNA clone IMAGE:4033352 5, mRNA sequence [CB559133]' |
| 'A_10_P012957' | 0,034632232 | -2,619152389 | 'gb\|dah92f02.x1 NICHD XGC Emb4 Xenopus laevis cDNA clone IMAGE:4957562 3, mRNA sequence [BI315005]' |
| 'A_10_P124274' | 0,010799053 | 2,614958169 | 'Unknown' |
| 'A_10_P255307' | 0,0071318 | 2,609487228 | 'tc\|Rep: MGC85021 protein - Xenopus laevis (African clawed frog), partial (86percent) [TC463026]' |
| 'A_10_P209688' | 0,036329533 | 2,60720232 | 'tc\|Rep: Periostin - Gallus gallus (Chicken), partial (6percent) [TC448413]' |
| 'A_10_P250632' | 0,018400836 | 2,602042088 | 'gb\|Xenopus laevis hypothetical LOC495490, mRNA (cDNA clone IMAGE:5516313), partial cds [BC085077]' |
| 'A_10_P109376' | 0,007287183 | -2,570214074 | 'gb\|AGENCOURT_73758169 NICHD_XGC_int_m Xenopus laevis cDNA clone IMAGE:8531400 5, mRNA sequence [EB481710]' |
| 'A_10_P074798' | 0,042087388 | -2,568524242 | 'gb\|BP720669 Osada Taira anterior neuroectoderm (ANE) pCS105 cDNA library Xenopus laevis cDNA clone XL446p05ex 3, mRNA sequence [BP720669]' |
| 'A_10_P170278' | 0,02366379 | -2,564005851 | ref\|Xenopus laevis 5-nucleotidase domain containing 3 (nt5dc3), mRNA [NM_001092501]' |
| 'A_10_P027126' | 0,035961148 | 2,549264901 | ref\|Xenopus laevis trefoil factor 1 (tff1-a), mRNA [NM_001085773]' |
| 'A_10_P037031' | 0,034906703 | -2,526580975 | ref\|Xenopus laevis aldehyde dehydrogenase 1 family, member A1 (aldh1a1), mRNA [NM_001087771]' |
| 'A_10_P085250' | 0,026971596 | -2,526266221 | 'gb\|AGENCOURT_89911475 NICHD_XGC_int_m Xenopus laevis cDNA clone IMAGE:8824591 5, mRNA sequence [EG583930]' |
| 'A_10_P233048' | 0,007672091 | -2,516360684 | 'tc\|Rep: UBXD2 protein - Xenopus laevis (African clawed frog), partial (5percent) [TC456324]' |
| 'A_10_P067057' | 0,012054382 | 2,514582483 | 'gb\|AGENCOURT_10486865 NICHD XGC Emb1 Xenopus laevis cDNA clone IMAGE:6635811 5, mRNA sequence [BU910077]' |
| 'A_10_P071725' | 0,03461937 | 2,510501046 | 'gb\|Xenopus laevis hypothetical protein LOC432287, mRNA (cDNA clone IMAGE:4202813), partial cds [BC072256]' |
| 'A_10_P004528' | 0,046211605 | -2,508002699 | ref\|Xenopus laevis RAB11 family interacting protein 5 (class 1) (rab11fip5), mRNA [NM_001091439]' |
| 'A_10_P004177' | 0,015106335 | 2,501176075 | 'ref\|Xenopus laevis uncharacterized protein MGC81332 (MGC81332), mRNA [NM_001091145]' |
| 'A_10_P220448' | 0,048199143 | 2,475042863 | 'Unknown' |
| 'A_10_P182973' | 0,008986014 | -2,474484909 | 'tc\|Rep: Dpf1 protein - Xenopus tropicalis (Western clawed frog) (Silurana tropicalis), partial (48percent) [TC438732]' |
| 'A_10_P090490' | 0,012263394 | -2,472969732 | 'gb\|DC134776 Yamamoto [DC134776]' |
| 'A_10_P004332' | 0,027880788 | -2,469094096 | ref\|Xenopus laevis orthodenticle homeobox 2 (otx2-b), mRNA [NM_001091486]' |
| 'A_10_P179013' | 0,003612953 | -2,455947426 | 'tc\|Rep: Secreted frizzled-related protein 2 - Xenopus tropicalis (Western clawed frog) (Silurana tropicalis), complete [TC437267]' |
| 'A_10_P009684' | 0,032289737 | -2,44053913 | 'gb\|db26g01.x1 Xenopus laevis oocyte Xenopus laevis cDNA clone XENOPUS_SOURCE_ID: 3, mRNA sequence [BE026446]' |
| 'A_10_P006302' | 0,014339947 | -2,425577595 | 'ref\|Xenopus laevis ribosomal protein L19 (rpl19-b), mRNA [NM_001093015]' |
| 'A_10_P031876' | 0,019367333 | 2,420974307 | 'ref\|Xenopus laevis thyroid hormone receptor beta-A (thrb-a), mRNA [NM_001096713]' |
| 'A_10_P222633' | 0,039821447 | -2,393951156 | 'Unknown' |
| 'A_10_P044266' | 0,04797818 | 2,349196995 | ref\|Xenopus laevis sarcoglycan, beta (43kDa dystrophin-associated glycoprotein) (sgcb), mRNA [NM_001112840]' |
| 'A_10_P037616' | 0,005617963 | -2,346433679 | 'ref\|Xenopus laevis ribonucleotide reductase M2 B (TP53 inducible) (rrm2b), mRNA [NM_001085900]' |
| 'A_10_P038586' | 0,01133829 | -2,343702564 | ref\|Xenopus laevis bagpipe homeobox (bapx), mRNA [NM_001085685]' |
| 'A_10_P160938' | 0,0262794 | 2,338733585 | 'tc\|Rep: Adipocyte-derived leucine aminopeptidase precursor (EC 3.4.11.-) (A- LAP) (ARTS-1) (Aminopeptidase PILS) (Puromycin-insensitive leucyl- specific aminopeptidase) (PILS-AP) (Type 1 tumor necrosis factor receptor shedding aminopeptidase regulator). -' |
| 'A_10_P185363' | 0,047250359 | 2,329245037 | 'tc\|Rep: Ketopantoate reductase ApbA/PanE - Exiguobacterium sibiricum 255-15, partial (5percent) [TC439595]' |
| 'A_10_P155993' | 0,031990681 | 2,308513447 | 'ref\|Xenopus laevis uncharacterized protein MGC115716 (MGC115716), mRNA [NM_001096096]' |
| 'A_10_P012969' | 0,008686601 | 2,30583177 | 'gb\|dah97b09.x1 NICHD XGC Emb4 Xenopus laevis cDNA clone IMAGE:4957769 3, mRNA sequence [BI315351]' |
| 'A_10_P159048' | 0,02271009 | 2,305751769 | 'Unknown' |
| 'A_10_P187178' | 0,007957127 | -2,295138896 | 'Unknown' |
| 'A_10_P003238' | 0,014822583 | 2,286112758 | 'ref\|Xenopus laevis cysteine conjugate-beta lyase 2 (ccbl2), mRNA [NM_001087271]' |
| 'A_10_P238003' | 0,046612948 | 2,283138391 | 'Unknown' |
| 'A_10_P216228' | 0,025588354 | -2,280240439 | 'tc\|Rep: Uncharacterized protein ENSP00000383185 - Homo sapiens (Human), partial (27percent) [TC450784]' |
| 'A_10_P013642' | 0,038472666 | -2,279178993 | 'gb\|DC012583 Osada Taira anterior endomesoderm (AEM) pCS105 cDNA library Xenopus laevis cDNA clone rxlk131i08 3, mRNA sequence [DC012583]' |
| 'A_10_P010631' | 0,00502126 | 2,274694212 | 'Unknown' |
| 'A_10_P137113' | 0,003489869 | -2,270926383 | 'Unknown' |
| 'A_10_P074175' | 0,011679398 | 2,259062981 | 'gb\|Xenopus laevis cDNA clone IMAGE:5572846, partial cds [BC070573]' |
| 'A_10_P081825' | 0,049067318 | 2,253969367 | 'gb\|BX850736 Wellcome CRC pcDNAI egg Xenopus laevis cDNA clone IMAGp998L148378 ; IMAGE:3431197 5, mRNA sequence [BX850736]' |
| 'A_10_P004883' | 0,012884044 | 2,250930561 | 'ref\|Xenopus laevis uncharacterized protein MGC80056 (MGC80056), mRNA [NM_001091745]' |
| 'A_10_P022851' | 0,020256138 | -2,238984165 | 'gb\|AGENCOURT_13321634 NICHD_XGC_Tad2 Xenopus laevis cDNA clone IMAGE:6872776 3, mRNA sequence [CB563920]' |
| 'A_10_P014797' | 0,028948953 | -2,237372048 | 'gb\|BJ091442 NIBB Mochii normalized Xenopus tailbud library Xenopus laevis cDNA clone XL097c16 3, mRNA sequence [BJ091442]' |
| 'A_10_P032791' | 0,039573572 | -2,229129811 | 'ref\|Xenopus laevis transmembrane tight junction protein claudin (cla), mRNA [NM_001088604]' |
| 'A_10_P239698' | 0,017659263 | -2,222811768 | 'Unknown' |
| 'A_10_P023129' | 0,005109742 | -2,220401922 | 'gb\|AGENCOURT_12983543 NICHD_XGC_Tad1 Xenopus laevis cDNA clone IMAGE:6877193 3, mRNA sequence [CB756059]' |
| 'A_10_P024542' | 0,026747188 | -2,220075722 | 'gb\|AGENCOURT_14239199 NICHD_XGC_Eye1 Xenopus laevis cDNA clone IMAGE:6957247 5, mRNA sequence [CD360966]' |
| 'A_10_P006380' | 0,004450576 | 2,217740977 | 'ref\|Xenopus laevis POU class 3 homeobox 4 (pou3f4), mRNA [NM_001100923]' |
| 'A_10_P000114' | 0,047891034 | -2,213572008 | 'ref\|Xenopus laevis hairy and enhancer of split 7, gene 1 (hes7.1), mRNA [NM_001088706]' |
| 'A_10_P171533' | 0,013087518 | -2,209772153 | 'tc\|Rep: La-related protein 7 - Xenopus tropicalis (Western clawed frog) (Silurana tropicalis), partial (6percent) [TC434477]' |
| 'A_10_P099341' | 0,048442878 | -2,204517444 | 'gb\|DC131709 Yamamoto [DC131709]' |
| 'A_10_P002714' | 0,033222362 | -2,204447651 | 'ref\|Xenopus laevis aryl-hydrocarbon receptor nuclear translocator 2 (arnt2), mRNA [NM_001087071]' |
| 'A_10_P271282' | 0,026114106 | -2,203960824 | 'tc\|Rep: Suc1-associated neurotrophic factor target XSNT - Xenopus laevis (African clawed frog), partial (27percent) [TC467487]' |
| 'A_10_P091754' | 0,049000527 | -2,199320852 | 'gb\|AGENCOURT_90759683 NICHD_XGC_int_m Xenopus laevis cDNA clone IMAGE:8823067 5, mRNA sequence [EG584719]' |
| 'A_10_P114451' | 0,040646945 | -2,199175318 | 'gb\|df95e09.x1 Xenopus laevis oocyte non normalized Xenopus laevis cDNA clone XENOPUS_SOURCE_ID:xlnnoc006i18 3 similar to TR:Q08701 Q08701 ZINC FINGER PROTEIN 5-2. ;, mRNA sequence [BG022020]' |
| 'A_10_P010399' | 0,036144353 | 2,19044608 | 'gb\|AGENCOURT_10509399 NICHD XGC OO1 Xenopus laevis cDNA clone IMAGE:6641567 5, mRNA sequence [BU914969]' |
| 'A_10_P050899' | 0,039853853 | 2,188473226 | 'gb\|AGENCOURT_10764728 Wellcome [CA971551]' |
| 'A_10_P122894' | 0,009226542 | 2,178634407 | ref\|Xenopus laevis lysophosphatidic acid receptor 2 (lpar2), mRNA [NM_001094167]' |
| 'A_10_P140368' | 0,046644184 | 2,172937726 | 'ref\|Xenopus laevis MGC81843 protein (MGC81843), mRNA [NM_001094395]' |
| 'A_10_P019842' | 0,038730021 | -2,169635179 | 'tc\|Rep: Immediate early response 5-like - Rattus norvegicus (Rat), partial (5percent) [TC462562]' |
| 'A_10_P043436' | 0,006473192 | 2,167719953 | 'ref\|Xenopus laevis AU RNA binding protein/enoyl-CoA hydratase (auh), nuclear gene encoding mitochondrial protein, mRNA [NM_001096053]' |
| 'A_10_P005084' | 0,031094072 | 2,165119225 | ref\|Xenopus laevis NIMA-related kinase 3 (nek3), mRNA [NM_001091706]' |
| 'A_10_P183353' | 0,011897726 | 2,143121895 | 'tc\|Rep: Elk3-prov protein - Xenopus laevis (African clawed frog), partial (52percent) [TC438870]' |
| 'A_10_P256807' | 0,017976268 | -2,139225864 | 'gb\|AGENCOURT_11285929 Wellcome [CA982460]' |
| 'A_10_P035276' | 0,007059215 | -2,135249786 | 'ref\|Xenopus laevis peptidase inhibitor 15 (pi15), mRNA [NM_001096301]' |
| 'A_10_P251242' | 0,041652219 | 2,13128958 | 'gb\|AGENCOURT_11113799 NICHD XGC Emb1 Xenopus laevis cDNA clone IMAGE:6863661 5, mRNA sequence [CA987095]' |
| 'A_10_P046266' | 0,013956422 | 2,129070567 | ref\|Xenopus laevis adiponectin receptor 1 (adipor1), mRNA [NM_001095969]' |
| 'A_10_P208418' | 0,002077929 | 2,12353892 | 'tc\|Rep: EF hand family protein - Tetrahymena thermophila SB210, partial (8percent) [TC447950]' |
| 'A_10_P202743' | 0,016221226 | 2,121782223 | 'tc\|Rep: MGC83759 protein - Xenopus laevis (African clawed frog), partial (61percent) [TC445868]' |
| 'A_10_P003515' | 0,020087279 | 2,10764679 | ref\|Xenopus laevis regulator of calcineurin 1 (rcan1), mRNA [NM_001086391]' |
| 'A_10_P000799' | 0,008847377 | -2,106266885 | 'gb\|BJ047180 NIBB Mochii normalized Xenopus neurula library Xenopus laevis cDNA clone XL015m07 3, mRNA sequence [BJ047180]' |
| 'A_10_P016436' | 0,030356566 | -2,102687156 | 'gb\|BP693411 Osada Taira anterior neuroectoderm (ANE) pCS105 cDNA library Xenopus laevis cDNA clone XL470h24ex 5, mRNA sequence [BP693411]' |
| 'A_10_P002023' | 0,017678389 | 2,099010765 | 'ref\|Xenopus laevis hematopoietic cell signal transducer A (hcsta), mRNA [NM_001085728]' |
| 'A_10_P177348' | 0,004529881 | 2,087126887 | ref\|Xenopus laevis phosphatase, orphan 2 (phospho2), mRNA [NM_001096935]' |
| 'A_10_P127504' | 0,011266896 | 2,073784041 | 'gb\|AGENCOURT_15209107 NICHD_XGC_Sp1 Xenopus laevis cDNA clone IMAGE:5506052 5, mRNA sequence [CF283032]' |
| 'A_10_P002672' | 0,019271303 | 2,066941155 | 'ref\|Xenopus laevis polymerase (RNA) III (DNA directed) polypeptide A, 155kDa (polr3a), mRNA [NM_001086833]' |
| 'A_10_P034401' | 0,045820711 | 2,06542641 | ref\|Xenopus laevis APG4A protein (apg4a), mRNA [NM_001112824]' |
| 'A_10_P022578' | 0,046729376 | -2,064674305 | 'gb\|AGENCOURT_13318418 NICHD_XGC_Tad1 Xenopus laevis cDNA clone IMAGE:6877869 3, mRNA sequence [CB561467]' |
| 'A_10_P206938' | 0,019904488 | 2,061997787 | 'tc\|Rep: Transmembrane protein 150 precursor. - Xenopus tropicalis, partial (7percent) [TC447401]' |
| 'A_10_P229223' | 0,031839634 | -2,060037819 | 'gb\|AGENCOURT_14161302 NICHD_XGC_Brn1 Xenopus laevis cDNA clone IMAGE:6953739 5, mRNA sequence [CD255092]' |
| 'A_10_P105700' | 0,039343096 | -2,057924072 | 'gb\|AGENCOURT_55791226 NICHD_XGC_FaB Xenopus laevis cDNA clone IMAGE:8070949 5, mRNA sequence [DT071674]' |
| 'A_10_P168283' | 0,014595142 | -2,057443573 | 'tc\|Rep: Acyl-CoA synthetase family member 2, mitochondrial precursor - Danio rerio (Zebrafish) (Brachydanio rerio), partial (25percent) [TC433245]' |
| 'A_10_P044831' | 0,030490155 | 2,056935552 | ref\|Xenopus laevis furry homolog (fry), mRNA [NM_001110757]' |
| 'A_10_P273727' | 0,019366212 | -2,056637911 | 'Unknown' |
| 'A_10_P159098' | 0,034167965 | -2,054284247 | gb\|Xenopus laevis morphogen (sonic hedgehog) mRNA, complete cds [L39213]' |
| 'A_10_P098156' | 0,032638281 | 2,045392585 | 'gb\|DC065464 Osada Taira anterior endomesoderm (AEM) pCS105 cDNA library Xenopus laevis cDNA clone xlk164e13 5, mRNA sequence [DC065464]' |
| 'A_10_P071150' | 0,035089685 | -2,044059934 | 'gb\|BJ627991 NIBB Mochii normalized Xenopus early gastrula library Xenopus laevis cDNA clone XL220b16 5, mRNA sequence [BJ627991]' |
| 'A_10_P034551' | 0,02334054 | 2,044016453 | 'ref\|Xenopus laevis galectin family xgalectin-IIa (xgalectin-IIa), mRNA [NM_001088735]' |
| 'A_10_P125654' | 0,02345845 | -2,040183882 | 'Unknown' |
| 'A_10_P001547' | 0,008841656 | -2,033857672 | 'gb\|DC051824 Osada Taira anterior endomesoderm (AEM) pCS105 cDNA library Xenopus laevis cDNA clone xlk150f03 5, mRNA sequence [DC051824]' |
| 'A_10_P257607' | 0,011419704 | -2,03278667 | 'Unknown' |
| 'A_10_P201743' | 0,017251137 | 2,032301185 | 'tc\|Rep: Sugar-phosphate isomerase, RpiB/LacA/LacB family - Ochrobactrum anthropi (strain ATCC 49188 / DSM 6882 / NCTC 12168), partial (12percent) [TC445492]' |
| 'A_10_P200113' | 0,003658316 | -2,032189667 | 'tc\|Rep: PREDICTED: calcium/calmodulin-dependent protein kinase IIA isoform 7 - Pan troglodytes, partial (11percent) [TC444863]' |
| 'A_10_P261007' | 0,044406153 | 2,029790612 | 'Unknown' |
| 'A_10_P034996' | 0,012373742 | 2,014092757 | 'ref\|Xenopus laevis MGC84453 protein (MGC84453), mRNA [NM_001094299]' |
| 'A_10_P110814' | 0,001534848 | -2,013361507 | 'gb\|Xenopus laevis hypothetical protein LOC432116, mRNA (cDNA clone IMAGE:5078910), partial cds [BC072375]' |
| 'A_10_P262417' | 0,021694162 | -2,008254042 | 'Unknown' |
| 'A_10_P086495' | 0,030437674 | 2,006799138 | 'gb\|AGENCOURT_77746069 NICHD_XGC_skin_m Xenopus laevis cDNA clone IMAGE:8641336 5, mRNA sequence [EB728416]' |
| 'A_10_P069492' | 0,008725345 | 2,00458556 | 'ref\|Xenopus laevis testis, prostate and placenta expressed (tepp), mRNA [NM_001172203]' |

## Supplementary table 2: Putative xWnt11 target genes
